# The Green Rebirth of Silicone Waste: Recent Advances in Recycling Technologies

**DOI:** 10.3390/ma19163392

**Published:** 2026-08-10

**Authors:** Guangxin Chu, Yifu Zhang, Zeyu Zheng, Chongtao Ding, Jingwen Xu, Shengping Yi, Jun Liao, Chi Huang

**Affiliations:** 1 College of Chemistry and Molecular Sciences, Wuhan University, Wuhan 430072, China; 2024282030190@whu.edu.cn (G.C.); yfzhang2023@whu.edu.cn (Y.Z.); 2016301040200@whu.edu.cn (Z.Z.); m13012411016@163.com (C.D.); 2024282030168@whu.edu.cn (J.X.); 2 Hubei Key Laboratory of Radiation Chemistry and Functional Materials, School of Nuclear Technology and Chemistry & Biology, Hubei University of Science and Technology, Xianning 437100, China

**Keywords:** silicone waste, physical recycling methods, chemical recycling methods, green chemistry

## Abstract

In silicone polymers, more than 90% of the main chain skeletons are linked by Si-O-Si bonds, with organic groups attached to the side chains and terminal groups. These polymers possess both organic and inorganic properties, exhibiting excellent resistance to high and low temperatures, good insulation, and weather resistance. They are widely used in aerospace, biomedical, electrical, transportation sectors and so on. Traditional methods for synthesizing silicones, such as carbon thermal reduction, have led to a sharp increase in global CO_2_ emissions. Consequently, developing technologies for the efficient degradation and recycling of small siloxane molecules from waste silicone polymers has become a research hotspot in the silicone industry. This paper explores the technical selection and research progress of physical recycling methods such as mechanical grinding, ultrasonication, and irradiation, as well as chemical recycling methods including pyrolysis and acid–base catalysis, in the field of silicone recycling. This holds significant practical implications for achieving a closed-loop cycle for silicones and reducing carbon emissions in the industry.

## 1. Introduction

**Figure 1:**
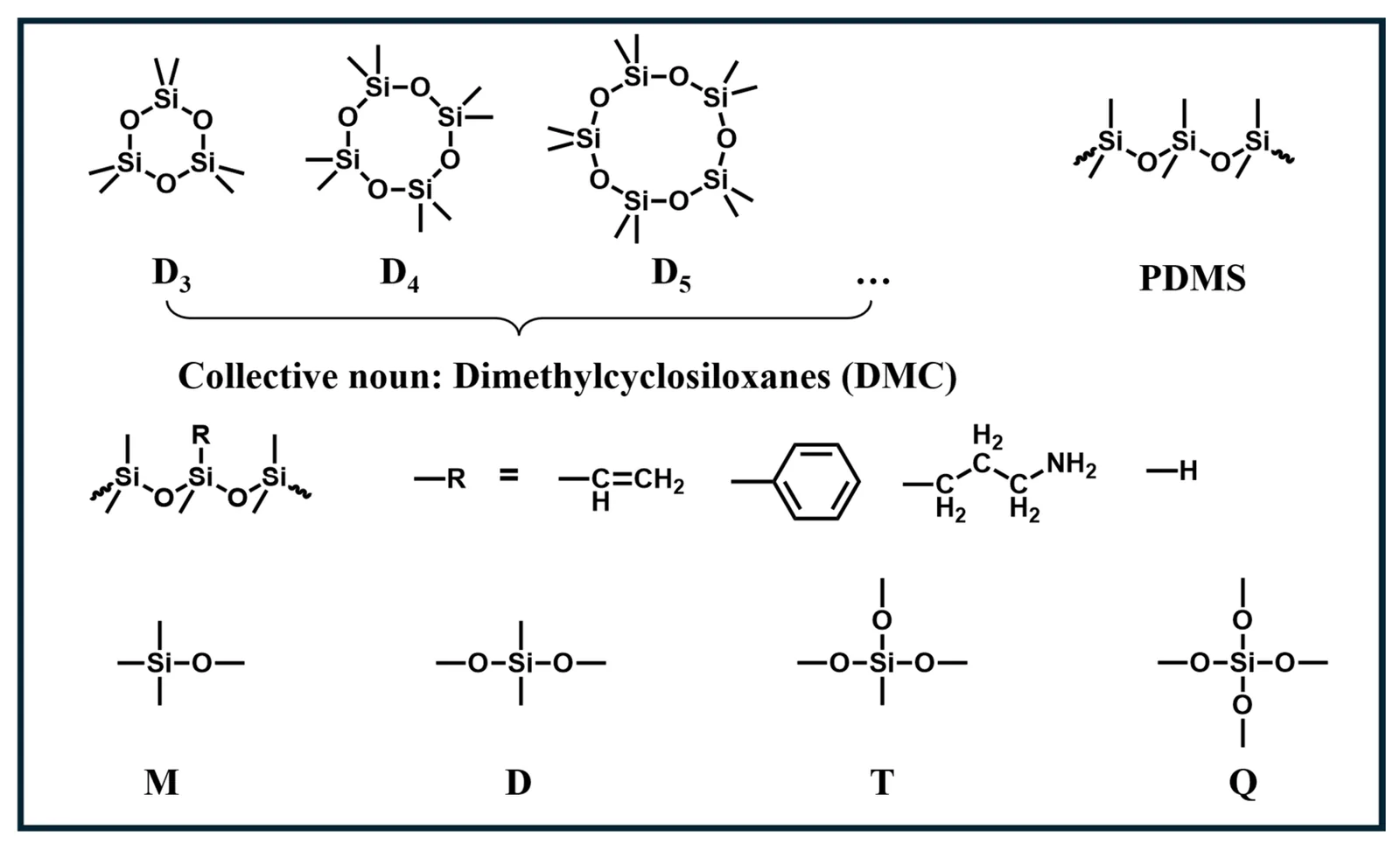
Representative chemical structures of silicone polymers [14].

In organosilicon chemistry [1], more than 90% of organosilicon polymers [2] possess main-chain backbones composed of Si-O-Si linkages [3]. As illustrated in Figure 1, the silicon atoms can bear various organic substituents [4], including alkyl [5], vinyl [6], aryl [7], aminopropyl [8], and silyl [9] groups, among others. Among these materials, polydimethylsiloxane (PDMS) is the most representative and widely used silicone polymer [10,11,12]. The bond dissociation energy of the Si-O bond in organosilicon polymers is approximately 4.62 × 10^2^ kJ·mol^−1^ [13], which is higher than that of C-C bonds in carbon-based organic compounds, approximately 3.50 × 10^2^ kJ·mol^−1^ [13], and C-O bonds, approximately 3.60 × 10^2^ kJ·mol^−1^. This high Si-O bond energy contributes to the excellent thermal stability of organosilicon materials. In addition, the Si-O-Si bond angle is approximately 145° [14], markedly larger than that of the C-O-C bond, approximately 110° [14], thereby imparting high flexibility to the siloxane backbone [15].

Silicone polymers exhibit considerable structural diversity because the silicon atom in each siloxane unit can be bonded to different numbers and types of organic substituents. According to the MDTQ nomenclature [13,16,17], siloxane units are generally classified into four fundamental structural units: monofunctional (M), difunctional (D), trifunctional (T), and quadrifunctional (Q) units. Through the combination and structural extension of these basic units, organosilicon materials with diverse physicochemical properties can be rationally designed and synthesized, including silicone oils [18,19,20], silicone rubbers [21,22,23], and silicone resins [24,25,26]. Moreover, the polarity [27], compatibility [28], crosslinking density [29], surface activity [30], and other properties of these materials can be precisely modulated by tailoring the type [31,32,33] and proportion [34,35,36] of organic side groups [37], as well as the nature of the end-capping groups [38,39,40]. Owing to this high degree of structural tunability, silicone-based materials show great potential for applications in fields with stringent performance requirements, including extreme environments [41], electronic devices [42], and biomedicine [43].

The global silicone industry has undergone rapid expansion [44,45] since the beginning of the 21st century. The silicone market is projected to increase from USD 22.9 billion in 2025 to USD 34.7 billion by 2034, corresponding to a compound annual growth rate (CAGR) of 5.3% during 2026–2034 [46,47]. In 2021, China’s silicon production was estimated to be approximately 60 million metric tons [48], making it the largest silicon producer worldwide. Among silicone products, linear polysiloxanes [49,50,51,52] represent one of the most important classes and are widely used as heat-transfer fluids [53], lubricants [54], and plasticizers [55], as well as key precursors for silicone rubber production. According to the vulcanization mechanism, silicone rubbers can be categorized into addition-cured silicone rubber [56], high-temperature-vulcanized silicone rubber [57], room-temperature condensation-cured silicone rubber [58], and related systems. Owing to their excellent physicochemical properties, these materials have been extensively applied in industrial fields, including vehicle manufacturing [59], composite insulators [60], and vibration-damping gaskets [61].

**Figure 2:**
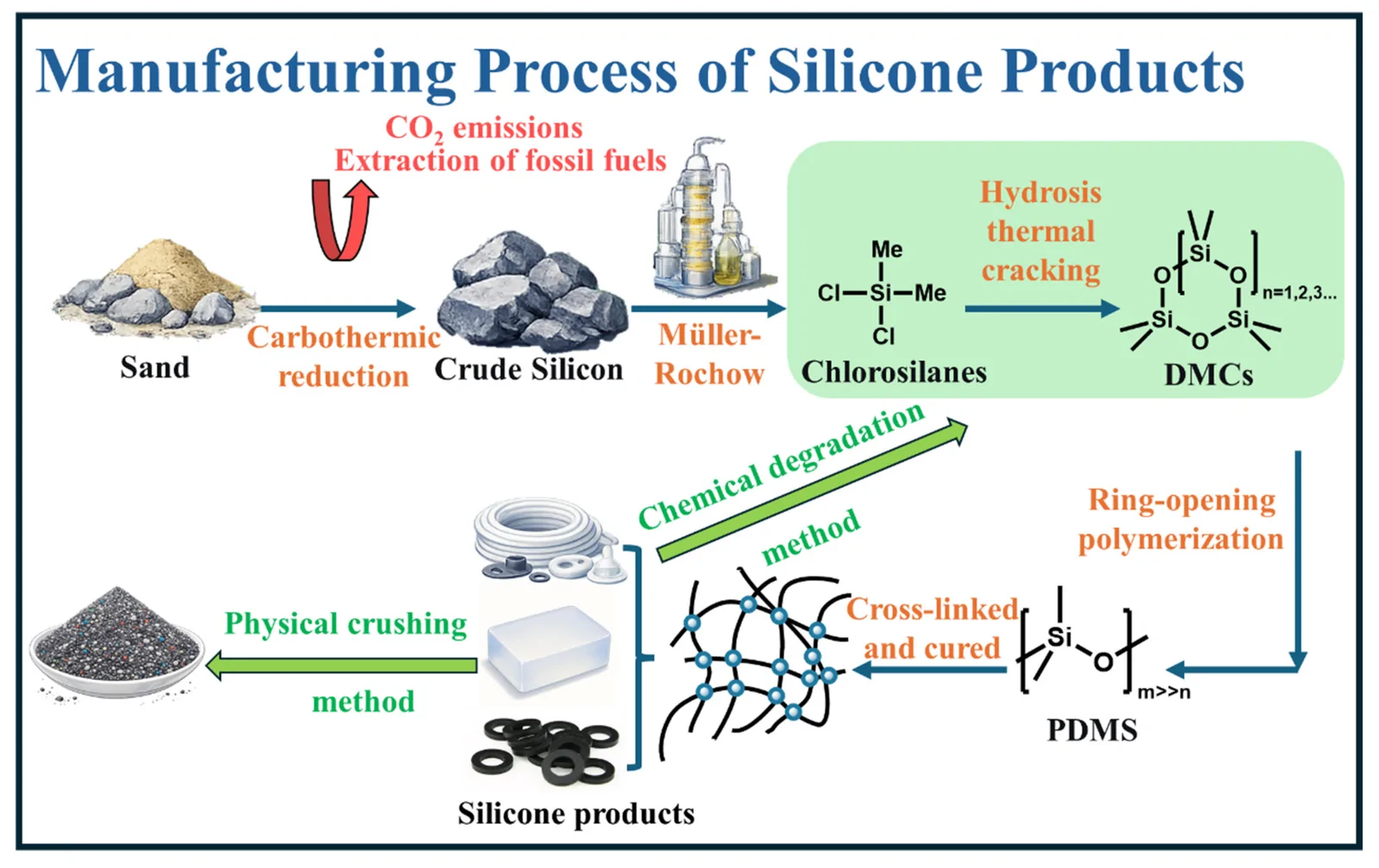
Schematic illustration of the manufacturing process for silicone products [73].

The production of silicone products is closely associated with carbon emissions [62], and Figure 2 schematically illustrates the manufacturing process of silicone products [46]. Metallurgical-grade silicon is typically produced by the carbothermal reduction of quartz sand with coke at approximately 2000 °C [63]. Subsequently, dimethyldichlorosilane is synthesized from silicon through the Roe-Müller process [64]. After hydrolysis and rearrangement, dimethylcyclosiloxane (DMC) is obtained and further used as a key intermediate for the synthesis of downstream silicone products. If silicone waste cannot be efficiently degraded and recycled for high-value utilization, the continuous production of silicone materials from primary resources will require substantial consumption of fossil fuels and lead to considerable CO_2_ emissions, thereby disrupting the carbon cycle and aggravating the greenhouse effect. Although silicone waste is not classified as a persistent organic pollutant (POP) [65,66] and can gradually degrade under natural conditions into products such as CO_2_, H_2_O, and SiO_2_ [14], this behavior differs fundamentally from that of conventional plastics such as polyvinyl chloride (PVC), which are difficult to depolymerize in the natural environment and may release toxic substances, including volatile organic compounds (VOCs), during degradation [67,68,69]. Nevertheless, the natural degradation capacity for silicone waste remains limited [70], and the degradation process is extremely slow [71,72]. Therefore, the development of efficient recycling technologies for silicone waste has become an important research focus in the silicone industry.

## 2. Recycling and Valorization of Waste Silicone Polymers

Current approaches for the recycling of silicone polymers are generally divided into physical and chemical recycling strategies [74,75,76,77,78,79,80,81,82,83,84,85]. Physical recycling methods primarily include mechanical comminution [75], gamma irradiation [86], electron-beam irradiation [87], and ultrasonic treatment [78], whereas chemical recycling mainly involves thermal cracking [88], acid-catalyzed depolymerization [89], and alkali-catalyzed depolymerization [83]. The references cited in this work encompass both seminal contributions and cutting-edge advances from the peer-reviewed literature indexed in the Web of Science, as well as representative patents that reflect commercial-scale emerging recycling technologies, ensuring a comprehensive coverage of fundamental principles, technological developments, and industrial applications.

### 2.1. Physical Recycling Methods

#### 2.1.1. Mechanical Comminution

**Figure 3:**
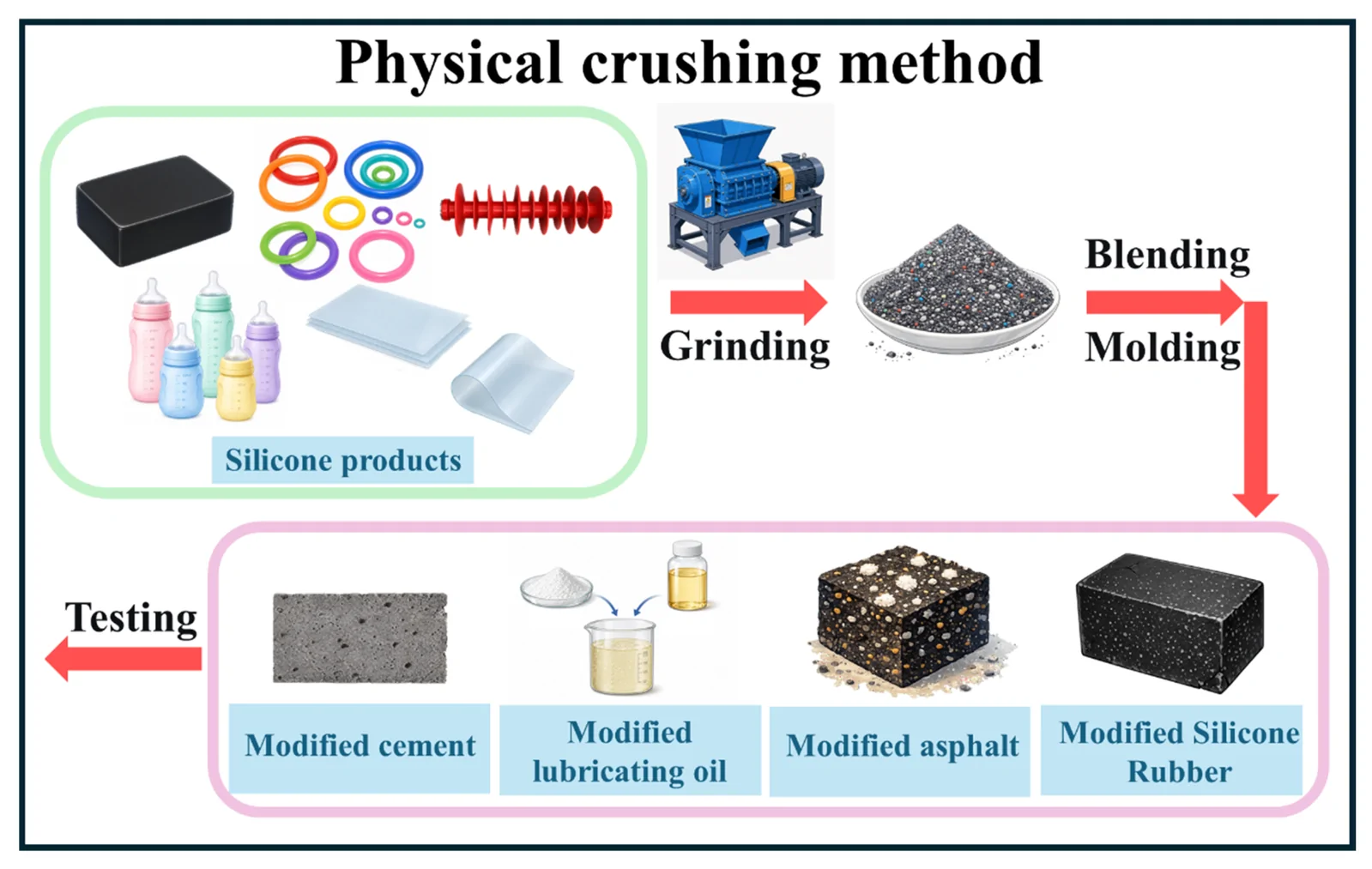
Schematic illustration of the mechanical comminution process for silicone rubber products [75,91,92,93].

Mechanical comminution is currently the simplest and most straightforward approach for the treatment of silicone waste. As shown in Figure 3, this process mainly relies on mechanical equipment, such as shearing machines, crushers, and ball mills, to reduce silicone waste into fine particles [90]. The obtained particles can be directly incorporated into polymer blends as modified fillers to fabricate recycled materials [91]. Alternatively, they can be subjected to secondary modification to improve their surface properties before being reused in the preparation of recycled materials [92]. When used as a dispersed elastomeric phase or functional filler, ground silicone rubber powder can potentially influence the toughness, impact resistance, damping performance, and interfacial compatibility of polymer blends. These effects depend largely on the particle size, dispersion state, filler content, and the type and density of surface-active functional groups on the silicone rubber particles. Appropriate particle-size control, uniform dispersion, and surface functionalization can significantly enhance interfacial adhesion between the silicone rubber particles and the polymer matrix, suppress phase separation, and consequently improve the mechanical strength and overall performance of the modified materials.

Hoffman et al. [75] incorporated silicone rubber powder with a particle size greater than 50 μm into high-temperature-vulcanized silicone rubber as a functional filler. The filler loading was varied from 0 to 60 phr. When the filler content reached 30 phr, the recycled silicone rubber exhibited a marked decrease in tensile strength and elongation at break. This deterioration was attributed to the absence of active functional groups on the surface of the silicone rubber powder, which differs from conventional reinforcing fillers such as SiO_2_. SiO_2_ can form strong interfacial interactions or covalent bonds with the polymer matrix, thereby enhancing adhesion and providing additional crosslinking sites. At a filler loading of 60 phr, compared with the unfilled sample, the compression set increased by 22%, whereas the maximum stress and elongation at break decreased by 45% and 25%, respectively.

Mayer et al. [94] reintroduced ground powder obtained from recycled liquid silicone rubber (LSR) into regenerated LSR. When the powder content reached 40%, the regenerated LSR could still be processed using conventional injection-molding equipment. Compared with the virgin LSR, the tensile strength and elongation at break of the regenerated material decreased slightly, by 16.6% and 12.6%, respectively. Nevertheless, the material largely retained its original performance, indicating favorable recyclability and potential cost advantages.

Building on the work of Mayer et al., Liedl et al. [90] further investigated the effects of Shore hardness, particle size, and powder loading on recycled LSR. Within the investigated ranges of Shore hardness of 30–70 HA, particle size of 0.2–1.0 mm, and powder loading of 10–30%, the mechanical properties of the recycled LSR remained relatively stable, and particle size showed no significant effect on the mechanical performance. Using LSR30, with a Shore hardness of 30 HA, as a model material, the Mullins effect associated with stress softening during tensile testing was further evaluated. In each tensile cycle, the specimen was stretched to 70% of its maximum strength. After ten cycles, the standard force decreased from 30 N to 22 N, while the reduction in standard force gradually diminished with increasing cycle number. (The Mullins effect refers to the stress-softening phenomenon observed in the mechanical response of filled rubbers, in which the stress–strain behavior depends on the maximum load previously experienced by the material, resulting in an instantaneous and irreversible softening response.)

Stiubianu et al. [95] incorporated silicone rubber powders derived from everyday products, including high-temperature-vulcanized gaskets, food-grade silicone, and silicone films, into PDMS raw rubber with a molar mass of 3.6 × 10^4^ g mol^−1^, followed by curing. The thermal stability, mechanical properties, and capacitive response of the resulting recycled silicone rubber were systematically characterized. Owing possibly to the absence of active functional groups on the surface of the silicone rubber powders, the elongation at break and tensile strength showed slight decreases, whereas Young’s modulus and thermal stability remained essentially unchanged. Moreover, the material exhibited a stable capacitive response under repeated cyclic loading, suggesting potential applications in soft robotics and medical devices.

In addition to their use as fillers in recycled silicone rubber, mechanically ground silicone rubber particles can also be blended with other materials, such as asphalt, cement mortar, and resins, for further modification. Qin et al. [91] ground waste silicone rubber from composite insulators into particles with a size range of 0.3–4.0 mm and incorporated them into cement mortar at volume fractions of 5–30% as partial substitutes for sand. Because of the relatively low rigidity of silicone rubber, the incorporation of silicone rubber particles reduced the compressive and flexural strengths of the cement mortar. However, when 15 vol% silicone rubber particles surface-modified with H_2_O_2_ or KOH were added, the mechanical properties of the cement mortar were improved. After 28 days of curing, the compressive strength of the H_2_O_2_- and KOH-modified composites increased by 10.56% and 12.44%, respectively, while the corresponding flexural strength increased by 1.82% and 7.27%, respectively. In addition, the thermal insulation and noise-reduction properties of the modified silicone rubber/cement composites were significantly enhanced.

Ullah et al. [92] employed a combination of waste silicone rubber crumb (CR) and sugarcane bagasse ash (SCBA) to modify the mechanical properties of asphalt concrete. The modified mixtures were evaluated by indirect tensile strength (ITS), Marshall stability, and flow value tests. The highest ITS was obtained with the incorporation of 4 wt% CR, based on the total mixture weight, and 25 wt% SCBA, based on the filler weight. In contrast, the maximum Marshall stability and the lowest flow value were achieved at 6 wt% CR and 25 wt% SCBA.

Gou et al. [74] incorporated waste silicone rubber powder (SRP) into resin-bonded friction materials (RFMs) at mass fractions of 0–20% to improve their frictional performance and vibration behavior. The results showed that SRP-modified RFMs inhibited crack initiation and propagation, facilitated the formation of a friction film, reduced fatigue wear, and enhanced wear resistance. At an SRP content of 10 wt%, the wear rate of the RFMs decreased by 29%, and the average friction-induced vibration amplitude decreased by 35.5%, corresponding to the optimal overall performance. However, excessive SRP loading induced phase separation, weakened the interfacial adhesion between the fibers and matrix, and consequently increased the vibration level.

Li et al. [96] employed the silane coupling agent (3-aminopropyl)triethoxysilane (KH550) to graft-activate the surface of waste silicone rubber particles. After activation, the surface morphology of the waste particles became rougher and more irregular, thereby enhancing their interfacial interaction with asphalt. This improved compatibility reduced the risk of cracking and enhanced the high-temperature stability and fatigue resistance of the asphalt.

Lee et al. [93] incorporated PDMS powder with an average diameter of 39 μm into a polyalphaolefin (PAO) lubricant to investigate the effects of PDMS powder and curing-agent contents on tribological properties. Within the PDMS powder loading range of 0–2.5 wt%, the lowest wear rate was achieved at a PDMS content of 2 wt%. The elasticity and recovery capability of the PDMS powder facilitated the formation of a lubricating film, enabling rapid energy dissipation and thus reducing wear. However, excessive PDMS powder loading caused nonuniform dispersion in the PAO lubricant, leading to disruption of the friction film and localized wear. Within the curing-agent content range of 0.1–1 wt%, increasing the curing-agent content enhanced the crosslinking density and mechanical strength of the PDMS powder, thereby promoting the formation of a more stable and durable friction film.

Mechanical comminution is relatively simple to operate and imposes low requirements on equipment and processing conditions. However, it also presents significant limitations in terms of application scope and recycling efficiency. In particular, mechanical grinding makes it difficult to precisely control particle morphology and surface roughness, rendering the resulting particles unsuitable for products with stringent performance requirements. In addition, the surfaces of the ground silicone rubber particles generally lack active functional groups [75], resulting in weak interfacial interactions when they are directly blended with materials such as silicone rubber or asphalt and consequently leading to phase separation [74]. Therefore, further surface modification [91,94] is typically required to enhance interfacial compatibility with the target matrix. During comminution, intense shear and compressive forces can partially disrupt the original three-dimensional crosslinked network of silicone rubber, increasing the number of dangling chains and free chain ends [97,98]. This structural damage consequently leads to deterioration in mechanical properties, such as tensile strength. In asphalt modification applications, once pulverized silicone rubber particles are incorporated into the asphalt matrix, the modified asphalt cannot be readily separated from the silicone rubber filler for subsequent recycling, resulting in poor recyclability and restricting its broader application.

#### 2.1.2. Irradiation and Ultrasonic Treatments

Among physical recycling methods, in addition to mechanical grinding, electron-beam irradiation, γ-ray irradiation, and ultrasonication can also be employed to degrade silicone polymers. Ultrasonication mainly relies on the cavitation effect [99,100,101], in which microbubbles rapidly grow and violently collapse within an extremely short period, generating high concentrations of excited-state reactive species in solution. Novel methods such as microwave-assisted [102] or supercritical fluid processes [78] primarily rely on physical pathways to achieve degradation of silicone polymers, and we therefore classify them under the broader category of physical recycling methods. These species can further induce changes in the physicochemical properties of polymers. Related techniques discussed above are generally unable to achieve complete degradation of silicone polymers. Instead, they primarily promote radical-mediated reactions, thereby inducing surface modification or aging of silicone rubber to improve its adaptability to extreme-temperature or radiation environments. With respect to polymer degradation, Price et al. [77,103] investigated the variation in the molecular weight of PDMS under ultrasonic conditions in 1996. In their study, a 1% (*w*/*v*) PDMS/toluene solution was subjected to ultrasonication at an intensity of 23 W·cm^−2^. The results showed that, during the initial stage of degradation, PDMS with a higher number-average molecular weight exhibited a faster decrease in molecular weight. If ultrasonication was continued, the number-average molecular weight eventually approached a limiting minimum value. However, PDMS with a minimum number-average molecular weight of 6.5 × 10^3^ showed no detectable molecular-weight change under ultrasonic treatment. In addition, the polydispersity index of the polymer (PDI = M_w_/M_n_) decreased during ultrasonication, indicating that the molecular-weight distribution gradually became narrower and tended toward a more monodisperse state.

Shim et al. [78] investigated the effect of water on the ultrasonic desulfurization of silicone rubber. Compared with dry desulfurization, ultrasonication in the presence of water generates hydroxyl radicals (OH·) and hydrogen radicals (H·) through the cavitation of water bubbles, thereby accelerating the cleavage of crosslinking sites and the scission of polymer-filler chemical bonds.

Zou et al. [102] conducted the alcoholysis of waste silicone rubber powders (SRPs) using vinyl-containing geraniol under microwave-assisted conditions. The nucleophilic attack of geraniol on Si-O-Si bonds introduced vinyl groups onto the surface of the rubber powders, thereby achieving surface modification. The modified rubber powders (gSRPs) were subsequently blended with commercial styrene-butadiene rubber (SBR). During vulcanization, the vinyl groups on the gSRP surface participated in the crosslinking reaction, improving the dispersion of the silicone rubber powders and strengthening interfacial adhesion within the rubber matrix. The resulting localized crosslinked structure endowed the gSRP-containing rubber with excellent damping properties, maintaining tanδ ≥ 0.3 over a broad temperature range of more than 80 °C. Although the degradation mechanism reported in this study is more closely related to chemical degradation, we mainly wanted to point out the helping effect of physical microwave irradiation in the process. Also, alcoholysis mainly works as a solvent-mediated reaction pathway. So, we put this study under physical recycling methods to show the role of microwave assistance.

Gamma rays [104] are high-energy photons that can generate electron–hole pairs [105] and undergo Compton scattering [106]. Upon absorption of γ-rays, organosilicon polymers generate free radicals [107]. These radicals can react with oxygen to form peroxy radicals [108,109], which further induce molecular rearrangement, oxidative reactions, and crosslinking in organosilicon polymers, thereby altering their chemical composition and physicochemical properties. Electron irradiation involves exposing silicone polymers to high-energy electron beams [110,111], which induces ionization and excitation of the polymer chains and releases orbital electrons to generate free radicals [112]. By regulating the irradiation dose and exposure time, the physicochemical properties of silicone polymers can be effectively modulated [113,114].

Deepalaxmi et al. [87] exposed silicone rubber/ethylene propylene diene monomer blends (SiR/EPDM) to electron-beam irradiation and γ-ray irradiation at a dose of 25 Mrad. Compared with the non-irradiated samples, both electron-beam-irradiated and γ-ray-irradiated SiR/EPDM blends exhibited increased electrical resistivity, tensile strength, and hardness, whereas their elongation at break decreased. Nevertheless, the elongation at break remained higher than 50% of that of the non-irradiated samples. After γ-ray irradiation, the contents of Si-H, C=C, and C-H bonds in SiR/EPDM increased. In contrast, electron-beam irradiation reduced the Si-H bond content and promoted crosslinking in SiR/EPDM. These results indicate that both electron-beam and γ-ray irradiation can accelerate the aging behavior of silicone rubber.

Min et al. [115] investigated silicone rubber sheets aged by thermal treatment and γ-ray irradiation. With increasing irradiation time and aging temperature, the high-frequency dielectric constant and ion concentration of the silicone rubber sheets increased, whereas the coefficient of thermal expansion decreased. These results suggest that oxidation occurred during irradiation, accompanied by an increased crosslinking degree of the silicone rubber and simultaneous Si-C bond cleavage. Surface potential decay measurements further revealed that crosslinking reactions dominated at low irradiation doses, whereas chain scission became the predominant process at high irradiation doses.

Ito et al. [86] examined the resistance of γ-ray-irradiated silicone rubber to water-vapor-induced degradation. In the FTIR spectra, the integrated absorption peak areas corresponding to Si-O-Si and Si-CH_3_ groups were higher for γ-ray-irradiated silicone rubber than for the unirradiated sample. In addition, ^29^Si NMR spectra showed enhanced signals associated with T and Q silicon units in the irradiated silicone rubber, indicating that γ-ray irradiation promoted crosslinking and generated additional crosslinking sites. After exposure to high-temperature steam, the unirradiated silicone rubber became brittle, whereas the γ-ray-irradiated silicone rubber exhibited improved resistance to steam degradation owing to its denser crosslinked network.

Wang et al. [116] irradiated silicone rubber with ultraviolet light, including UVA at 340 nm and UVB at 313 nm, and extracted and concentrated the silicone oligomers generated from the irradiated silicone rubber using a Soxhlet extractor. The concentrated extracts contained cyclic siloxanes D_3_-D_19_ and linear siloxane oligomers L_8_-L_17_. In the extract obtained after UVA irradiation, the content of small-ring cyclic siloxanes D_3_-D_9_ remained lower than that of D_10_-D_19_ throughout the irradiation period of 0–1110 h. This phenomenon was attributed to the further involvement of highly strained small-ring molecules in crosslinking reactions, which is referred to as the “post-curing” of silicone rubber. In contrast, in the extract obtained after UVB irradiation, the initial content of small-ring cyclic siloxanes D_3_-D_9_ was lower than that of D_10_-D_19_; however, after 670 h of irradiation, the D_4_ content increased markedly. This increase was ascribed to the cleavage of PDMS chain segments in silicone rubber induced by prolonged UVB irradiation. Therefore, UVB irradiation may involve two competing processes: post-curing and chain scission.

Wang et al. [117] treated silicone rubber coatings with oxygen plasma, also referred to as atomic oxygen (AO), and ultraviolet (UV) irradiation. Their results showed that AO-treated coatings exhibited a higher hydroxyl content and a larger number of crosslinking sites than UV-treated coatings, accompanied by more extensive cleavage of C-H and Si-C bonds. The aging effects induced by AO and UV irradiation in silicone rubber mainly manifested as crosslinking reactions involving the polymer side chains, resulting in reduced surface flexibility and elongation at break, as well as increased modulus values.

Ultrasonic treatment [77], γ-ray irradiation [115], electron-beam irradiation [87], plasma treatment [117], and UV irradiation [117] can generate abundant free radicals, thereby effectively promoting surface crosslinking reactions in silicone rubber and inducing aging or surface modification. However, these methods do not enable complete degradation of silicone polymers or effective separation and recovery of fillers. The resulting modified rubber powders and coatings are therefore mainly used in applications requiring resistance to extreme temperatures, irradiation, or other harsh environmental conditions.

### 2.2. Chemical Recycling Methods

#### 2.2.1. Thermal Cracking

**Figure 4:**
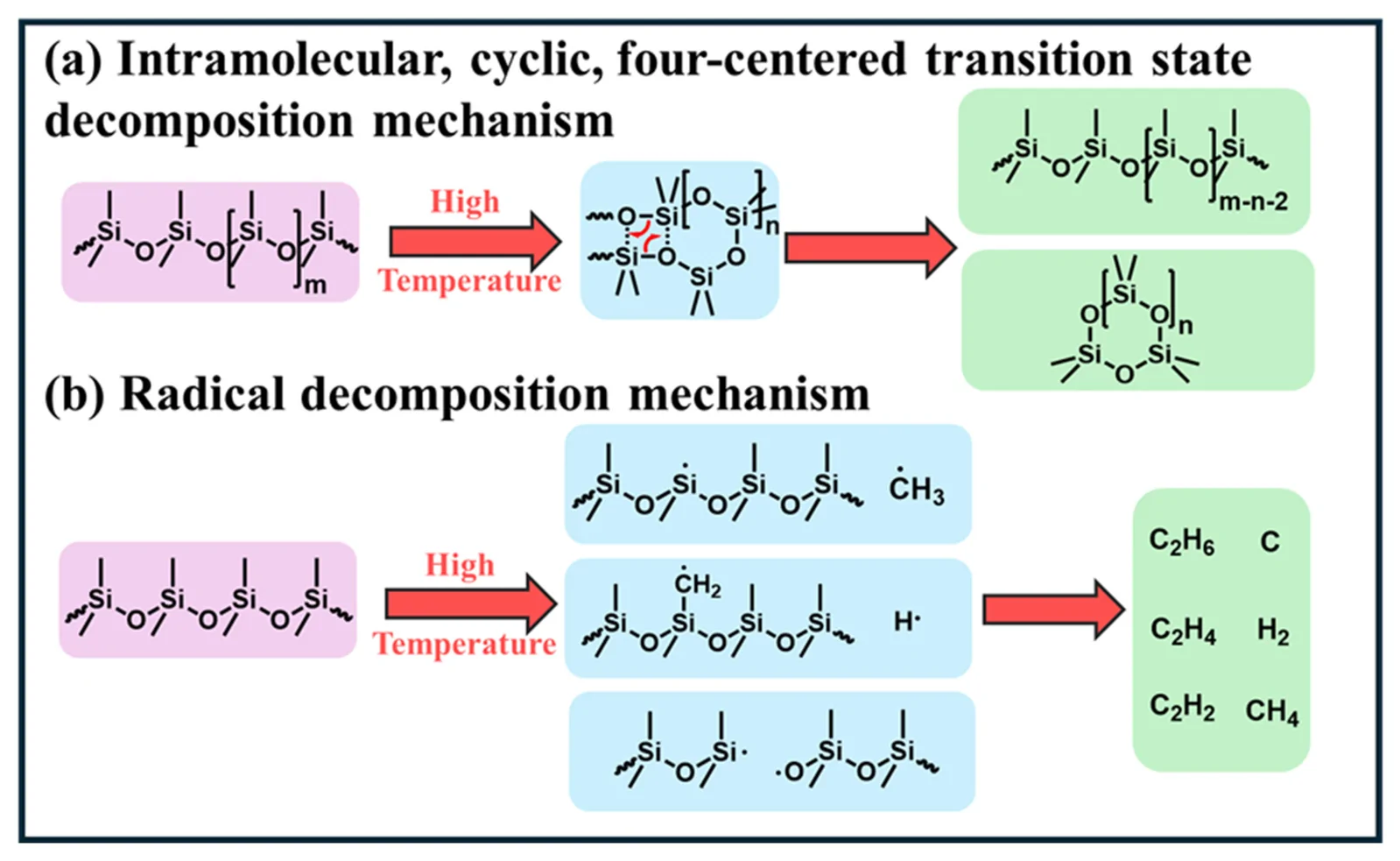
Reaction pathways of PDMS under high-temperature pyrolysis conditions: (**a**) cyclization reaction, (**b**) radical reaction [119].

Research on pyrolysis dates back to the 1940s [118]. Initially, pyrolysis was mainly used to evaluate the thermal stability of materials, but it has gradually developed into one of the earliest and most established approaches for material degradation and recovery. As illustrated in Figure 4, using PDMS as a representative example [119], the high-temperature pyrolysis of silicone polymers involves two competing reaction pathways. One pathway is the intramolecular cyclization of the PDMS backbone through the formation of a tetracoordinate cyclic transition state, which mainly leads to backbone depolymerization and the formation of cyclic siloxanes, such as dimethylcyclosiloxanes (DMCs). The other pathway involves side-chain cleavage, accompanied by the generation of free radicals. This classical theory has been further substantiated by subsequent theoretical developments and experimental evidence. Advances in chromatographic techniques have enabled the identification of decomposition products associated with the proposed reaction pathway, while theoretical calculations have provided additional support for the validity of the theory.

Patnode et al. [118] first investigated the pyrolysis of PDMS in 1946. Under pyrolysis conditions of 350–400 °C, atmospheric pressure, and a N_2_ atmosphere, hexamethylcyclotrisiloxane (D_3_) and octamethylcyclotetrasiloxane (D_4_) accounted for 44% and 24% of the pyrolysis products, respectively. These results preliminarily demonstrated that thermal cracking can convert PDMS into cyclic dimethylsiloxane compounds.

Andrianov et al. [80] investigated the thermal decomposition of PDMS bearing terminal hydroxyl (-OH), sodium silanolate (-ONa), potassium silanolate (-OK), and sulfonic acid (-SO_3_H) groups under vacuum at temperatures below 550 °C. They concluded that Si-O bond cleavage was initiated by the terminal groups. Kucera et al. [120,121,122] further demonstrated that the depolymerization mechanism of hydroxyl-terminated PDMS differs below and above 430 °C. At temperatures above 350 °C, hydroxyl groups not only initiate Si-O bond cleavage but also promote Si-C bond scission.

Around 1970, Rode et al. [81] found that the terminal groups of PDMS significantly influence the depolymerization behavior of the siloxane backbone. Upon heating, hydroxyl-terminated PDMS, bearing reactive terminal groups, first undergoes end-initiated back-biting at the hydroxyl sites, with depolymerization occurring at 350–370 °C. In contrast, methyl-terminated PDMS, which contains relatively inert terminal groups, undergoes depolymerization at 390–420 °C, indicating its higher thermal stability.

Thomas et al. [123] investigated the thermal depolymerization of methyl-terminated PDMS with different molecular weights under vacuum at 420 °C. The results showed that the thermal decomposition products of PDMS were mainly low-molecular-weight cyclic siloxanes ranging from D_3_ to D_11_. This process involved the formation of an intramolecular four-membered cyclic transition state and Si-O bond rearrangement. The activation energy for thermal depolymerization was found to be independent of molecular weight. Specifically, the activation energy for depolymerization of the PDMS backbone was approximately 40 kcal mol^−1^, which is less than half of the Si-O bond energy, approximately 108 kcal·mol^−1^. The much lower activation energy compared with the Si–O bond energy indicates that PDMS depolymerization is unlikely to proceed via direct random cleavage of Si–O bonds, but rather through a lower-energy intramolecular rearrangement or back-biting pathway to form cyclic siloxanes. This finding suggests that PDMS depolymerization is primarily governed by molecular structure and reaction kinetics. It has been proposed that silicon atoms in the polymer backbone can participate in the formation of energetically favorable transition states, thereby enabling low-energy depolymerization pathways. In contrast, conventional carbon-chain polymers do not undergo analogous depolymerization pathways as readily because of their different electronic structures.

Grassie et al. [124] investigated the depolymerization of PDMS under vacuum and collected the resulting cyclic oligomers in a cold trap. Cyclic oligomers were detected at 343 °C, and the thermal decomposition rate reached a maximum at 443 °C. In contrast, the decomposition temperature of PDMS containing 5% KOH decreased by approximately 250 °C, accompanied by the formation of gaseous CH_4_, which could not be captured by the cold trap. In the same year, Grassie et al. [125] examined the thermal cracking of hydroxyl- and methyl-terminated polymethylphenylsiloxane (PMPS) under a N_2_ atmosphere. The results showed that hydroxyl-terminated PMPS exhibited higher thermal stability than methyl-terminated PMPS, which was opposite to the behavior observed for PDMS. This difference may be attributed to the role of Si-OH groups in promoting Si-Ph bond cleavage, thereby inducing premature decomposition and generating various cis and trans isomers of cyclomethylphenylsiloxane. In addition, Grassie and co-workers conducted related studies on the thermal decomposition of poly(dimethyl-methylphenylsiloxane) [126], poly(dimethyl-diphenylsiloxane) [127], and phenylene–polysiloxane block copolymers [128,129,130,131].

Mantz et al. [132] investigated the pyrolysis behavior of cage-type polysiloxanes, namely polyhedral oligomeric silsesquioxanes (POSS), and POSS-siloxane block copolymers. Under atmospheric pressure in a N_2_ atmosphere, the hydroxyl-free compounds C_y6_Si_6_O_9_, C_y8_Si_8_O_12_, and C_y8_Si_8_O_11_(OSiMe_3_)_2_ began to sublime at 359, 463, and 360 °C, respectively. Their peak sublimation temperatures were associated with molecular weight and structural symmetry. In contrast, the hydroxyl-containing compound C_y8_Si_8_O_11_(OH)_2_ sublimed in two stages, at 230–450 °C and 450–650 °C. The first stage was attributed to the sublimation of POSS macromolecules, whereas the second stage involved the formation of hydrocarbons such as cyclohexane and cyclohexene. Cyclohexane may be generated through Si-C bond cleavage, while cyclohexene may form via β-elimination. The pyrolysis of POSS-siloxane copolymers occurred mainly between 390 and 650 °C, with D_3_ and D_4_ cyclic siloxanes detected in the range of 390–518 °C. Compared with the initial depolymerization temperature of pure PDMS, approximately 340 °C [124,133,134], the steric hindrance imposed by POSS cages partially inhibited the depolymerization and cyclization of siloxane segments. At 525–650 °C, cyclohexyl-derived hydrocarbons were the predominant products, whereas small amounts of CH_4_ and H_2_ were detected at temperatures above 650 °C.

Cao [135] employed online pyrolysis-gas chromatography/mass spectrometry (Py-GC/MS) to qualitatively and quantitatively analyze the pyrolysis products of waste silicone rubber insulators over the temperature range of 300–600 °C. The dominant reaction was the cyclization of the silicone rubber backbone, leading to the formation of cyclic siloxanes. With increasing temperature, the pyrolysis products gradually shifted toward lower-membered cyclic siloxanes, mainly D_3_ and D_4_, accompanied by a decrease in the content of inorganic residues. Notably, the D_3_ content increased from 1.7% to 78.8%, indicating enhanced selectivity toward smaller cyclic products at elevated temperatures. Similar results were reported by Jiang et al. [136] for condensation-cured and addition-cured silicone rubbers pyrolyzed at 600 °C. For condensation-cured silicone rubber, the pyrolysis products were mainly D_3_-D_6_ cyclic siloxanes, with D_3_ accounting for 82%. In contrast, addition-cured silicone rubber primarily produced D_3_-D_10_ cyclic siloxanes, with a D_3_ content of 60%, along with trace amounts of Si-H and vinyl-containing cyclic siloxanes.

Wen et al. [79] subjected room-temperature condensation-cured silicone rubber to temperature cycling between −25 and 70 °C for 20, 40, and 60 days. Hydrophobicity migration tests showed that, with prolonged temperature cycling, additional migration pathways for low-molecular-weight silicone oils were formed, facilitating their migration to the surface of the rubber matrix and thereby increasing the contact angle. Meanwhile, compared with untreated silicone rubber, the temperature-cycled silicone rubber exhibited a higher residual weight at 700 °C, and longer cycling durations further increased the residual residue. This improvement may be attributed to additional crosslinking reactions induced by temperature cycling. Similar results were reported by Schneider et al. [137], who subjected two commercial silicone rubbers, RTV615 and Sylgard 184, to temperature cycling between −40 and 150 °C. They concluded that temperature cycling promoted densification and aging of the polymer network through tighter crosslinking, resulting in a higher elastic modulus in the aged silicone rubber than in the unaged material [138].

Ren et al. [139] synthesized silicone oil containing B-O-Si linkages and sulfoxide groups through block copolymerization using boronic acid, thionyl chloride, and dimethyldichlorosilane, and investigated its thermal degradation behavior under a N_2_ atmosphere. Compared with hydroxyl-containing silicone oil, the initial weight-loss temperature (T_5_) and the maximum weight-loss temperature (T_max_) during pyrolysis increased by 105 °C and 173 °C, respectively. The incorporation of phenylboronic acid and sulfoxide groups effectively enhanced the thermal stability of the modified silicone oil. In particular, the large steric hindrance of these groups suppressed intramolecular cyclization reactions and inhibited Si-O-Si bond cleavage along the silicone oil backbone.

Hu et al. [76] investigated the pyrolysis behavior of high-temperature-vulcanized silicone rubber by combining solid-state shear grinding with KOH-assisted treatment. Compared with the original rubber powder obtained by simple mechanical crushing, the rubber powder treated by shear grinding and KOH blending exhibited a higher dissolution rate in Soxhlet extraction with cyclohexane. During pyrolysis, the initial weight-loss temperature (T_5_) and the maximum weight-loss temperatures (T_p1_ and T_p2_) decreased by 44.34 °C, 4.99 °C, and 163.15 °C, respectively. These results suggest that mechanical stress and chemical degradation exert a synergistic effect, effectively shortening the pyrolysis time and producing pyrolysis products with a narrower distribution.

Wang et al. [119] employed density functional theory (DFT) calculations to elucidate the mechanism of radical Si-C bond cleavage during the high-temperature pyrolysis of PDMS, as well as the formation pathways of gaseous products, such as CH_4_ and H_2_, liquid products, including dimethylcyclosiloxane (DMC) cyclic compounds, and solid products, such as carbon black. The methyl and hydrogen radicals generated through the concurrent cleavage of Si-C and C-H bonds can undergo rearrangement and recombination to form small-molecule gases, including H_2_, CH_4_, and C_2_H_6_. Under prolonged high-temperature conditions, C_2_H_6_ further undergoes dehydrogenation and carbonization, ultimately leading to the formation of carbon black. During the thermal cracking of organosilicon polymers, depolymerization of the Si-O-Si backbone in siloxane segments, cleavage of side-chain Si-C bonds, and radical reactions occur simultaneously and are difficult to control at elevated temperatures. Consequently, in addition to DMC cyclic compounds, byproducts such as H_2_, CH_4_, C_2_H_6_, and carbon black are generated, which limits the yield of high-value DMC products.

Bi et al. [140] developed a cascade thermochemical reaction process for the high-temperature steam cracking of PDMS fluid in a tube furnace to produce hydrogen-rich syngas. At a steam-to-feed ratio of 2.0, a carbon conversion of 75.9% was achieved, with a maximum syngas yield of 42.23 mmol.g^−1^ at 900 °C. The resulting residue was subsequently reacted with carbon black through a Joule-heating flash vaporization process to produce SiC materials. The synthesized SiC material contained graphene-like structures that provided capillary channels for H_2_O transport. In addition, SiC could bind to the graphene surface to form cation-transport channels, thereby facilitating charge transfer. As a result, the SiC-graphene composite exhibited higher electrical responsivity than conventional graphene.

Vo et al. [141] investigated the pyrolysis behavior of two-component addition-cured PDMS elastomers with different mixing ratios, denoted as E_1_ = 5:1, E_2_ = 10:1, E_3_ =15:1, and E_4_ = 20:1, under N_2_ and O_2_ atmospheres. Under a N_2_ atmosphere, the decomposition of E_1_-E_4_ proceeded in three stages: 430–480 °C, 480–600 °C, and 600–750 °C. The slight weight loss in the first stage was mainly attributed to the conversion of Si-H bonds to Si-OH bonds, followed by coupling reactions between Si-H and Si-OH groups and dehydration condensation among Si-OH groups. The rapid weight loss in the second stage was primarily associated with intramolecular cyclization of the polymer segments, whereas the slight weight loss in the third stage resulted from the cleavage of side-chain Si-CH_3_ bonds, generating small molecules such as H_2_ and CH_4_. At 800 °C, the residual weights of E_1_, E_2_, E_3_, and E_4_ were 50.0%, 37.2%, 32.2%, and 19.3%, respectively. The higher crosslinking degree suppressed intramolecular cyclization of the elastomer chains during the second stage, thereby increasing the residual weight, while the decomposition behavior in the first and third stages remained largely unchanged. Under an O_2_ atmosphere, the residual weight of E_2_ at 800 °C was higher than that under N_2_. This was attributed to the generation of oxygen radicals during pyrolysis, which increased the number of crosslinking sites in the elastomer and consequently enhanced the residual weight.

Wu et al. [142] synthesized organosilica aerogels through acid-catalyzed dehydration condensation of polymethylmethoxysilane (PMMS) with dimethoxysilanes bearing different organic substituents, including methyl, vinyl, and phenyl groups. They further investigated the pyrolysis behavior of these aerogels over the temperature range of 30–800 °C. The solid residue yields of the methyl-, vinyl-, and phenyl-substituted aerogels were 35.7%, 64.6%, and 71.3%, respectively, with corresponding average activation energies of 41.0, 79.5, and 116.8 kJ·mol^−1^. The higher bond dissociation energies of vinyl and phenyl substituents increased the energy barriers for bond cleavage. Meanwhile, their larger steric hindrance suppressed intramolecular cyclization reactions, thereby inhibiting the pyrolysis of the aerogels.

Thermal cracking does not require the use of catalysts, thereby avoiding issues related to catalyst recovery and regeneration. However, this advantage is accompanied by harsh reaction conditions, which typically involve high temperatures, non-atmospheric pressure, and a protective gas atmosphere, leading to relatively high energy consumption and environmental burdens. In addition, thermal pyrolysis often generates a large number of by-products. Besides DMC cyclic oligomers, gaseous low-molecular-weight hydrocarbons and carbonaceous residues are also formed [119], resulting in relatively low recovery efficiency and resource utilization.

#### 2.2.2. Acid-Catalyzed Depolymerization

Acid-catalyzed depolymerization [14,48,143,144] primarily proceeds through coordination between the lone-pair electrons of oxygen atoms in the siloxane backbone and the electron-deficient centers of Brønsted or Lewis acids. This interaction increases the electrophilicity of adjacent silicon atoms, rendering them more susceptible to nucleophilic attack by oxygen atoms. The formation of cyclic products is typically accompanied by side reactions, including condensation, chain transfer, and chain extension. Consequently, at equilibrium, a mixture of cyclic siloxanes, such as D_3_, D_4_, and D_5_, together with low-molecular-weight silicone oils, is obtained. Common Brønsted acids [48,145] include H_2_SO_4_, HNO_3_, HF, and trifluoromethanesulfonic acid, whereas representative Lewis acids [73,89,146] include FeCl_3_, ZnCl_2_, AlCl_3_, BF_3_·OEt_2_, and GaCl_3_. When capping agents, such as halogen-containing reagents bearing F or Cl, or alkoxy-containing reagents, are introduced into the reaction system in stoichiometric amounts beyond those required for catalysis (often several times the molar amount of Si-O bonds), PDMS can be completely converted into silane monomers, including chlorosilanes, fluorosilanes, and alkoxysilanes.

**Figure 5:**
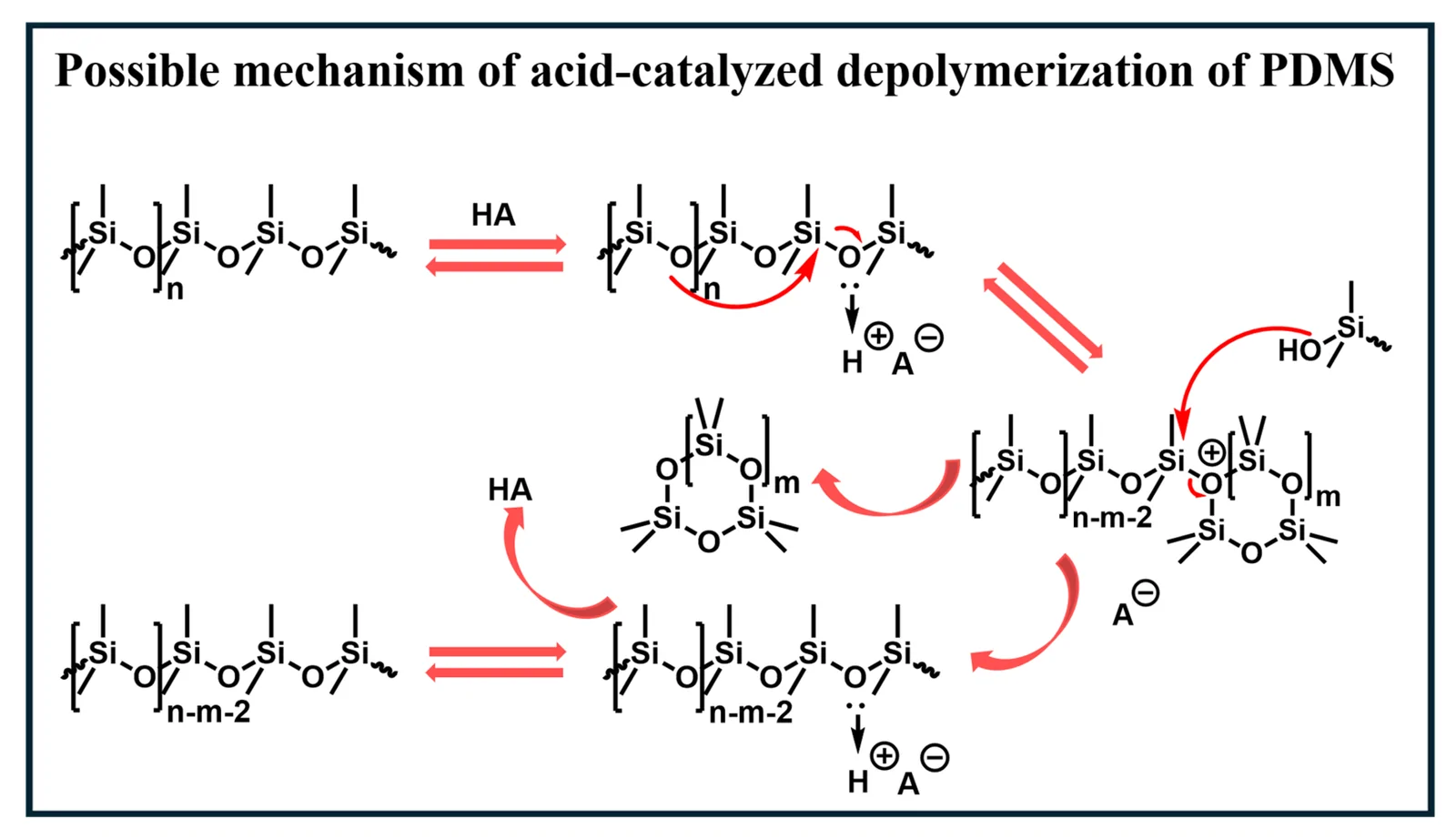
Proposed mechanism of acid-catalyzed depolymerization of PDMS [46,147].

Hurd et al. [147] proposed that the mechanism of acid-catalyzed depolymerization of organosilicon polymers differs substantially from that of base-catalyzed depolymerization. In the acid-catalyzed process, H^+^ first coordinates with the oxygen atom in the siloxane backbone, followed by cleavage of the siloxane bond to generate a silanol species and either a siloxane-derived ionic intermediate or a silyl halide species. The active hydroxyl group is considered an important intermediate that catalyzes the rearrangement and cleavage of PDMS. The equilibrium rate and product distribution depend strongly on the concentrations of silanol groups and siloxane-derived ionic species. Salahudeen et al. [48] proposed a possible mechanism for acid-catalyzed depolymerization, as illustrated in Figure 5.

Chiang et al. [148] further supported Hurd’s interpretation. The molecular weight of silicone oil A, which was prepared using concentrated sulfuric acid followed by extensive rapid washing, tended to stabilize as the α value, defined as the ratio of D_4_ to end-capping groups, increased. In contrast, the molecular weight of silicone oil B, prepared using 70% sulfuric acid followed by slow washing, increased with increasing α value. Silicone oil A likely formed sulfonate-terminated groups; however, extensive washing further diluted the acid concentration, thereby weakening hydrolytic condensation and resulting in only minor changes in molecular weight. Conversely, silicone oil B was prepared by the slow addition of water in the absence of solvent, which allowed prolonged contact between the silicone oil and sulfuric acid. This promoted a higher degree of condensation and consequently led to a further increase in molecular weight.

Apblett et al. [149] found that, at elevated temperatures, Al(CH_3_)_3_ can react with cyclic siloxanes, (RMeSiO)_x_ (R = Me, n-C_18_H_37_, -CH_2_CH_2_CF_3_, or Ph), leading to siloxane bond cleavage and the formation of dimeric aluminosiloxanes. Single-crystal X-ray diffraction analysis provided the unit-cell parameters of [Me_2_Al(OSiMe_2_Ph)]_2_: space group P2_1_/n, a = 7.970(3) Å, b = 24.563(10) Å, c = 13.322(4) Å, and β = 105.05(3)°. Muhaupt et al. [150] obtained similar results by reacting Al(CH_3_)_3_ with dimethylcyclosiloxane or PDMS at 175 °C. X-ray crystallographic analysis of [Me_2_Al(OSiMe_3_)]_2_ gave the following unit-cell parameters: space group C2/m, a = 111.350(5) Å, b = 13.101(2) Å, c = 6.918(4) Å, and β = 111.60(2)°.

Alexander et al. [151] reacted AlCl_3_ with dimethyl silicone grease at a 1:1 ratio in hexane and found that AlCl_3_ could cleave Si-O bonds, producing a large number of [(ClSiMe_2_OAlCl_2_)]_2_ crystals. Compared with Al(CH_3_)_3_, AlCl_3_ contains longer Al-Cl bonds, and the bridging ability of chloride ions may be sterically hindered by methyl groups. Therefore, they proposed that AlCl_3_-catalyzed depolymerization is more likely to proceed through a conventional four-center cyclic transition-state mechanism.

Chojnowski et al. [152] argued that, compared with base-catalyzed processes, the mechanism of cationic ring-opening polymerization of organosilicon compounds is considerably more complex and has not yet been fully elucidated. Using cyclotrisiloxane as a model compound, they demonstrated that its acid-catalyzed ring-opening polymerization in the presence of trifluoromethanesulfonic acid involves the formation of a terminal cyclotrisiloxonium ion intermediate. Ring formation proceeds through the reaction between the oxonium ion intermediate and the terminal hydroxyl group of another polymer chain, accompanied by regeneration of the acid catalyst during the intermolecular reaction. This interpretation is highly consistent with the conclusions proposed by Hurd et al. [147].

Okui et al. [153] degraded block copolymers consisting of poly(tetramethylphenylene) segments and PDMS segments using a 48% HF solution at 30 °C. They found that HF preferentially attacked the amorphous Si-O bonds in the flexible PDMS segments, whereas the Si-O bonds adjacent to the rigid benzene-ring-containing poly(tetramethylphenylene) segments were scarcely affected. After 40 h of degradation, the crystallinity of the polymer increased from 20% to 98%, indicating that most of the PDMS segments had been successfully degraded.

Feng et al. [154] investigated the degradation behavior of addition-cured silicone rubbers with different hardness values in acidic aqueous solutions. The results showed that increased hardness and acidity led to more pronounced weight loss of the silicone rubber, accompanied by greater leaching of calcium-containing fillers. In the FTIR spectra, the intensities of the Si-O-Si absorption bands at 1080 and 1010 cm^−1^ decreased significantly, indicating that the Si-O-Si bonds were cleaved under proton attack. Meanwhile, decomposition of the silicon-bonded side groups generated additional crosslinking sites, resulting in a slight increase in the elastic modulus.

Zhang et al. [145] subjected crushed waste silicone rubber to pyrolysis in a vacuum reactor and collected the pyrolysis liquid using a separation device, thereby enabling effective separation of inorganic fillers from the polymer matrix. Under optimized conditions of 8 mL concentrated sulfuric acid, a reaction temperature of 300 °C, and a reaction time of 50 min, 20 g of waste silicone rubber powder was pyrolyzed, achieving the highest liquid yield. The selectivities toward D_4_, D_5_, and D_6_ in the pyrolysis products were 38.56%, 42.72%, and 11.67%, respectively.

Xu et al. [155] prepared an SO_4_^2−^/Al_2_O_3_-TiO_2_ composite solid superacid catalyst by an impregnation method. The catalyst was reacted with 400 g of waste silicone rubber powder under mechanical stirring at 130 °C for 4 h in a reactor. After 7 h of reaction, the reaction was terminated, and the cracking residues were discharged while hot. Subsequently, 0.5 g of a 1 mol·L^−1^ nitric acid solution was added to the distilled crude DMC product (approximately 250 g). The mixture was heated to 120 °C and allowed to react for 2 h, after which 0.5 g of a 1 mol·L^−1^ aqueous NaOH solution was added to terminate the reaction. After distillation and subsequent separation, approximately 250 g of DMC cyclic compounds were obtained. Among these products, D_4_ accounted for approximately 146 g, followed by about 24 g of D_5_, 30 g of D_6_, and 10 g of D_7_ and higher-membered cyclic siloxanes.

Gao et al. [156] prepared a modified diatomite catalyst from concentrated sulfuric acid and diatomaceous earth. Silicone rubber scraps were co-ground with SiO_2_ and FeSO_4_, followed by the addition of 1–1.5 wt% modified diatomite and 0.3–0.5 wt% sulfonic acid to catalyze the cracking of the silicone rubber scraps. Under vacuum at 120–150 °C, 97.1–98.1% of DMC cyclic compounds were recovered, and the resulting residue could be further converted into silica after high-temperature treatment.

Yang et al. [157] prepared a rare-earth catalyst based on strontium aluminate doped with europium oxide. After reaction with a silicone rubber suspension at 120–150 °C for 3–5 h, followed by phase separation, dehydration, and vacuum distillation, DMC cyclic compounds were obtained in yields corresponding to 70–90% of the mass of the waste silicone rubber.

Enthaler et al. [89] utilized the Lewis acidity of zinc salts for the cleavage of siloxane bonds. Using 5.0 mol% Zn(OTf)_2_ and benzoyl fluoride as a fluorine source at a threefold molar excess relative to the substrate, hydroxyl-terminated silicone oil (M_n_ = 550 g·mol^−1^) was converted into fluorosilanes in yields of up to 99%. Excellent cleavage efficiency was also achieved for other organosilicon substrates. The Lewis acidic zinc salt activates the Si-O bond and lowers the reaction energy barrier, while the benzoic anhydride generated during the reaction can be regenerated using KF. In the same year, Enthaler et al. [146] employed more cost-effective iron salts for polysiloxane degradation. The iron salts reduced the dissociation energy of the Si-O bond by 79.5 kJ·mol^−1^, whereas zinc salts reduced it by only 33.9 kJ·mol^−1^ under the same conditions. (These data were obtained from density functional theory (DFT) calculations.)

Peter Döhlert et al. [158,159,160] employed boron trifluoride diethyl etherate (BF_3_·OEt_2_) as both a Lewis acid catalyst and a fluorinating reagent to convert methyl silicone oil, hydroxyl-terminated silicone oil, and branched polysiloxanes into difluorodimethylsilane monomers. BF_3_·OEt_2_ provides an electron-deficient boron center that coordinates with the lone-pair electrons of oxygen atoms in the polysiloxane backbone, while simultaneously serving as a fluorine source for end-capping. The boron-containing byproducts can be regenerated into BF_3_ in the presence of NaF/H_2_SO_4_, thereby enabling catalyst recycling. When hydroxyl-terminated silicone oil (M_n_ = 550 g·mol^−1^) was depolymerized using 300 wt% BF_3_·OEt_2_, a fluorosilane yield of 93% was achieved at 100 °C within 1 h. For elastomers containing T and Q units, depolymerization yields exceeding 70% were also obtained. In a subsequent study, Döhlert et al. [160] further extended the BF_3_·OEt_2_ system to the depolymerization of polydimethylsilazane, affording dimethyldifluorosilane in 92% yield under reaction conditions of 120 °C for 3 h.

Forens et al. [161] investigated the depolymerization of PDMS and various silicone elastomers using 4-dodecylbenzenesulfonic acid. Under ambient conditions, the molecular weight decreased progressively as the reaction time increased from 0 to 7 days. The final molecular weight could be regulated by adjusting the amounts of acid and end-capping agent. Using this approach, they successfully prepared silicone oils containing vinyl and Si-H functional groups, which could be further used to synthesize addition-cured silicone rubber, thereby enabling closed-loop recycling.

Lindström et al. [162] prepared a metal-free silicone rubber based on hydroxymethyl-modified PDMS and polysilazane. In a tetrahydrofuran solution containing 0.055 mol·L^−1^ acetic acid at 40 °C, the crosslinking sites in the silicone rubber could be cleaved by acetic acid, converting the network into a liquid depolymerized product. This depolymerized material could subsequently be recured with polysilazane. When the regenerated silicone rubber was blended with the original silicone rubber at a 1:1 ratio and vulcanized, the resulting material retained essentially comparable mechanical properties.

Vu et al. [73] employed the Lewis acid GaCl_3_ as a catalyst and excess BCl_3_ as a chlorine source. Under mild conditions of 40 °C for 30 min, PDMS was converted into dimethyldichlorosilane in a yield exceeding 99%. BCl_3_ and GaCl_3_ acted synergistically to enable an almost barrierless chlorine-transfer process. The formation of GaCl_4_^−^ increased the positive charge density on adjacent silicon atoms, inducing a structural transformation of the silicon center from tetrahedral to trigonal-pyramidal geometry and facilitating the formation of a silane-like cationic intermediate. This configuration promoted the attack of GaCl_4_^−^ on silicon atoms, thereby assisting the conversion of Si-O bonds into Si-Cl bonds and increasing the reaction rate by approximately 10^6^-fold.

In acid-catalyzed methods [13], Brønsted acids generally require lower catalyst loadings; however, the increase in acid concentration during the later stages of the reaction can accelerate equipment corrosion. In addition, the recovered products often consist of mixtures of DMC cyclic compounds and linear or branched oligomers [154], which complicates downstream separation. Lewis acid catalysis has emerged as one of the current research hotspots in this field. Lewis acid systems enable the recovery of halosilanes and alkoxysilanes under milder conditions. Nevertheless, chlorine and fluorine sources used as capping agents are typically required in 2–3-fold excess [89,146,158], and the recovered halosilanes or alkoxysilanes can generate substantial amounts of byproducts, such as hydrohalic acids or alcohols, during repolymerization.

#### 2.2.3. Alkali-Catalyzed Depolymerization

Base-catalyzed depolymerization [13,46,48] primarily proceeds through nucleophilic attack on silicon atoms in the siloxane backbone, resulting in Si-O bond cleavage. In contrast to conventional organic polymers, whose backbones are mainly composed of C-C or C-O bonds, organosilicon polymers possess Si-O-Si backbones. Although carbon and silicon both belong to Group 14 of the periodic table and can form four σ bonds, silicon can adopt hypercoordinate structures during reaction processes, which facilitates nucleophilic substitution at the silicon center [13,163,164]. Common Brønsted bases used for this process include NaOH [165], KOH [166], and N(CH_3_)_4_OH [167], whereas representative Lewis bases include KF [168], CsF [169], and tetrabutylammonium fluoride (TBAF) [170]. New recycling methods such as ionic liquid/deep eutectic solvent-mediated approaches and the use of solid catalysts like TBAF or KF/Al_2_O_3_ primarily exploit the Lewis basicity and small steric hindrance of F^−^ to achieve efficient degradation of silicone polymers. These methods are included in this study within the broader scope of alkali-catalyzed depolymerization.

**Figure 6:**
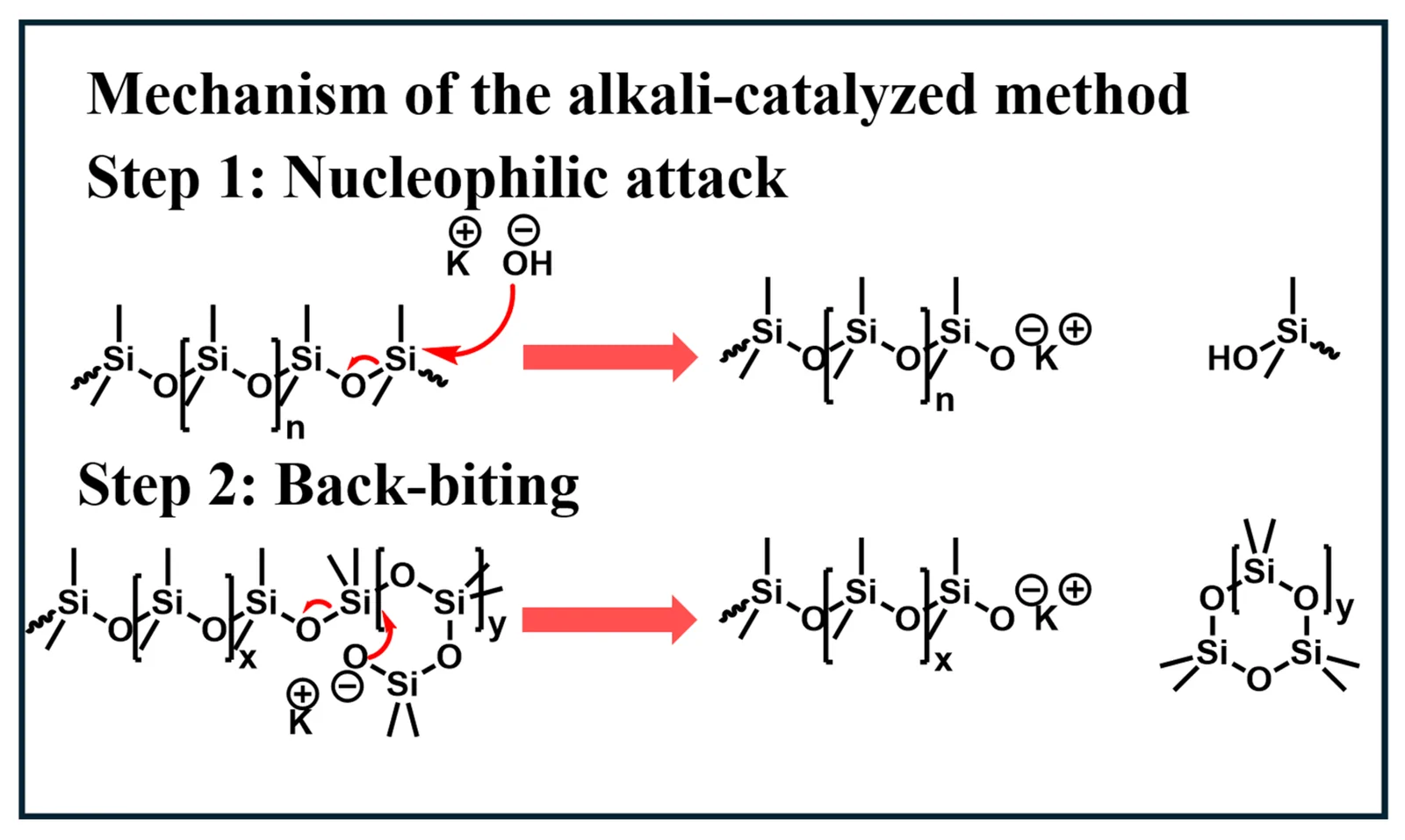
Schematic illustration of the alkali-catalyzed depolymerization mechanism of PDMS [46,171].

Taking KOH-catalyzed PDMS cracking as an example [13,48,171], the proposed mechanism of alkali-catalyzed depolymerization is schematically illustrated in Figure 6. Dissociated OH^−^ first attacks the silicon atom in the siloxane backbone, leading to Si-O bond cleavage and the formation of a silanolate species and a polysiloxane chain bearing an active silicon center. Driven by the reaction equilibrium, the silanolate anion subsequently undergoes an intramolecular back-biting reaction, producing dimethylcyclosiloxane (DMC) cyclic compounds and shorter silanolate-terminated chains. Under equilibrium conditions at atmospheric pressure, the ratio of DMC cyclic compounds to PDMS is approximately 15:85 [172]. This ratio is primarily governed by thermodynamic equilibrium. Increasing the reaction temperature or continuously removing the generated DMC under vacuum shifts the equilibrium toward back-biting and DMC formation, thereby promoting continuous depolymerization of PDMS chains. Compared with acid-catalyzed depolymerization, the reaction mechanism of base-catalyzed processes is better established. Cyclic compounds are generally considered to form predominantly through the intramolecular back-biting of silanolate anions. Subsequent mechanistic studies and experimental investigations into the base-catalyzed degradation of organosilicon polymers have provided further support for this mechanism.

Kučera et al. [171] investigated the anionic ring-opening polymerization of D_4_. In an aqueous KOH solution at 150 °C, the molecular weight of the resulting polysiloxane decreased, indicating significant depolymerization. Furthermore, the rate of anionic ring-opening polymerization of D_4_ was affected by both the solvent and the basicity of the alkali catalyst. Solvents such as anisole and diphenylamine can form pentacoordinate silicon complexes with silicon atoms, thereby slowing the polymerization rate. When the alkali catalyst was changed from KOH to NaOH and then to LiOH, the catalyst basicity decreased, and the polymerization rate declined markedly. The study further suggested that the active center responsible for anionic ring-opening polymerization also serves as the active site for depolymerization. From a mechanistic perspective, the anionic ring-opening polymerization of cyclic siloxanes is not merely a chain-growth process, but rather a reversible process governed by competition between chain propagation and intramolecular backbiting. Chain propagation promotes the formation of linear polysiloxanes, whereas intramolecular backbiting results in the formation of cyclic siloxanes. Therefore, the ring-opening polymerization of cyclic siloxanes and the depolymerization of linear polysiloxanes essentially represent the forward and reverse directions, respectively, of the same reversible equilibrium system.

Okamoto et al. [173] prepared a KF/Al_2_O_3_ solid catalyst by supporting KF on Al_2_O_3_ and applied it to the depolymerization of MM and D_3_ in the presence of dimethyl carbonate. The highest yields of trimethylmethoxysilane and dimethyldimethoxysilane reached 85% and 94%, respectively. When a small amount of methanol was added during the depolymerization of MM, the yield of trimethylmethoxysilane increased to over 98%. In a subsequent study, Okamoto et al. [168] further demonstrated that the depolymerization rate depended on the type of alcohol but was independent of the type of dialkyl carbonate. After polarization of the Si-O bond by the KF/Al_2_O_3_ catalyst, methanol attacks the silicon center, leading to Si-O bond cleavage and formation of the corresponding monomer and water. The generated water subsequently reacts with dimethyl carbonate to regenerate methanol; therefore, dimethyl carbonate acts as the apparent depolymerization reagent. Using the KF/Al_2_O_3_ solid catalyst and dimethyl carbonate in methanol, various substrates, including poly(methylphenyl-dimethyl)siloxane and poly(methylvinyl-dimethyl)siloxane, were successfully depolymerized to afford mixtures of methylphenyldimethoxysilane, methylvinyldimethoxysilane, and dimethyldimethoxysilane. Meanwhile, the SiO_2_ reinforcing filler was also converted into tetramethyl orthosilicate.

Yu et al. [174] employed a composite catalyst composed of a Schiff base and hydroxide to depolymerize waste silicone rubber under a N_2_ atmosphere at 140–200 °C, producing dimethylcyclosiloxane cyclic compounds. The lone-pair electrons on the nitrogen atom of the C=N bond in the Schiff base, in combination with hydroxide, enhanced the stability of the reaction system, lowered the required pyrolysis temperature, significantly reduced the hydroxide dosage, and improved the depolymerization efficiency.

Feng et al. [175] used organosilicon halogenating agents, such as trimethylchlorosilane and dimethyldichlorosilane, to depolymerize PDMS and peroxide-cured silicone rubber. The addition of a small amount of FeCl_3_ catalyst accelerated the reaction, enabling the depolymerization of PDMS and silicone rubber with a molecular weight of 6.0 × 10^5^ into organosilicon oligomers with a viscosity of only approximately 25 mPa·s.

According to an earlier German patent (DE 2961698, 1979), as summarized by Xing et al. [165], the pyrolysis of 300 g of vulcanized silicone rubber use 750 g of di-n-butylamine (Bu_2_NH) as the reaction solvent and 2.5 wt% tetramethylammonium hydroxide (N(CH_3_)_4_OH) as the catalyst. The addition of a second catalyst charge of 7.5 wt% further increased the conversion, and high-purity DMC cyclic compounds were successfully recovered from waste silicone rubber through fractional and vacuum distillation.

Oku et al. [166] depolymerized room-temperature-vulcanized silicone rubber under vacuum using toluene as the solvent. The yield of dimethylcyclosiloxane cyclic compounds initially increased and then decreased with increasing KOH loading. When the molar ratio of KOH to Si-O bonds in the polymer reached 0.08, the DMC yield reached a maximum of 65%. At catalyst loadings below 0.08, the DMC yield increased with increasing catalyst content, mainly because the larger number of catalytic active sites accelerated the depolymerization reaction. However, when the catalyst loading exceeded 0.08, the DMC yield decreased with further increases in KOH content. This decrease was attributed to the formation of potassium bis(silanolate) species. In these species, the low electrophilicity of silicon in Si-O^−^K^+^ moieties suppresses the back-biting reaction. In addition, excessive catalyst loading promotes the formation of gel-like substances, leading to a sharp decrease in DMC yield. When weak acids, such as potassium dihydrogen phosphate (KH_2_PO_4_) and potassium hydrogen terephthalate (KOOCC_6_H_4_COOH), were used to partially neutralize potassium bis(silanolate) species and convert them into potassium monosilanolate species, additional DMC cyclic compounds could be continuously distilled during the secondary reaction. In the same year, Oku et al. [167] used a methanol/diethylamine/hexane mixed solvent system to simultaneously convert filler-containing high-temperature-vulcanized silicone rubber into a polymer solution and separate fumed silica. After heating to remove quaternary ammonium residues from the filler surface, relatively pure fumed silica was obtained. The polymer solution was then subjected to vacuum depolymerization with a small amount of heat-resistant KOH catalyst, achieving a monomer yield of up to 84%.

Brook et al. [176] etched the surfaces of silicone elastomers using trifluoromethanesulfonic acid, TBAF, and KOH. When the catalyst-containing solvent was unable to effectively penetrate the interior of the elastomer, the etching reaction occurred predominantly on the elastomer surface. Under acid-catalyzed conditions, slow and uniform etching was observed, increasing the surface roughness from 15 to 125 nm. In contrast, when KOH penetrated into the crosslinked network of the elastomer, the surface roughness increased to as high as 850 nm. The etching behavior of TBAF was strongly dependent on the solvent. No etching occurred in dry methanol, diethylene glycol dimethyl ether, or acetone, whereas etching proceeded in tetrahydrofuran, producing a surface with moderate roughness.

Protsak et al. [82] employed dimethyl carbonate to depolymerize PDMS and PMPS. The effective depolymerization of PDMS and PMPS by dimethyl carbonate was confirmed by ^1^H NMR spectroscopy, size-exclusion chromatography, and viscosity monitoring. They further developed a surface functionalization strategy by introducing dimethyl carbonate into partially depolymerized polysiloxanes. After modification with PMPS/dimethyl carbonate, the SiO_2_ particles became smaller, and the modified polysiloxane species were uniformly distributed on the SiO_2_ surface, resulting in improved hydrophobicity and thermal stability.

Krug et al. [83] found that a small amount of F^−^ could dissolve in highly cured silicone resin at 250 °C. Although the cured silicone resin was insoluble in tetrahydrofuran, trace F^−^ induced equilibrium rearrangement of the T and Q units in the silicone resin, rendering the resin soluble in tetrahydrofuran at room temperature. Upon solvent removal, the crosslinked network was re-formed. This method primarily exploits the unique properties of fluorine-containing quaternary ammonium ionic liquids, which can provide a high concentration of sterically unhindered nucleophilic fluoride ions. As a result, Si–O bonds can be cleaved under mild reaction conditions.

Rupasinghe et al. [170] used TBAF to depolymerize polyphenylmethylsiloxane and found that the yield and selectivity of the depolymerization products were strongly dependent on the solvent. Depolymerization was negligible in protic solvents, such as water and methanol, and in nonpolar solvents, such as toluene. Only limited depolymerization occurred in dipolar solvents, including acetonitrile and dichloromethane. In contrast, complete depolymerization was achieved in tetrahydrofuran, affording cyclomethylphenylsiloxane in a yield exceeding 95%.

Weitkamp et al. [177,178] exploited the weakly coordinating nature of phosphonium hydroxide salts to achieve rapid depolymerization of organosilicon polymers at room temperature. In the presence of only 0.1 mol% catalyst, PDMS was rapidly depolymerized at room temperature into a cyclic siloxane mixture containing 2% D_3_, 82% D_4_, and 8% D_5_.

Warner et al. [179] employed fluorinated coordinating ionic liquids, such as tetrabutylammonium difluorotriphenylsilicate (TBAT), and fluorinated ionic liquids, such as tetrabutylammonium fluoride (TBAF), for the depolymerization of PDMS fluids. GPC analysis showed a decrease in polymer molecular weight, accompanied by a transition of the system from a gel-like state to a liquid state. However, TBAT exhibited a weaker depolymerization capability than TBAF, which could convert PDMS into small molecules or linear oligomers.

Vu et al. [180] applied a complex formed from potassium trimethylsilanolate and 18-crown-6 to the depolymerization of silicone polymers, thereby effectively enhancing the nucleophilicity of the reactive species. Under mild conditions of 60–170 °C, this system catalyzed the depolymerization of PDMS and afforded DMC cyclic compounds in high yields. For various waste silicone oils on a 100 g scale, depolymerization yields of approximately 98–99% were achieved, and the catalyst maintained good activity over five consecutive cycles.

Deller et al. [181] investigated the depolymerization of commercial silicone gel using various combinations of alkali metal fluorides, including K^+^, Na^+^, Ca^2+^, and Cs^+^ salts, with polyethylene glycol or 18-crown-6. After static incubation for 24 h at ambient temperature and pressure under sealed conditions, linear polyethers such as PEG failed to depolymerize the commercial silicone gel. In contrast, in the 18-crown-6 system, slight discoloration of the silicone gel was observed after prolonged reaction. Although the degradation degree under these conditions was limited, these observations provided preliminary evidence for the feasibility of using crown ethers in the catalytic depolymerization of organosilicon compounds.

Chu et al. [169] employed a K[(cryptand [2.2.2])]F complex for the degradation of PDMS. Host-guest recognition of K^+^ enhanced F^−^ solubility and nucleophilicity. In DMSO and n-octadecanol, the yield exceeded 95% with higher than 99.5% selectivity for dimethylcyclosiloxanes cyclic compounds. The catalyst remained highly active over ten consecutive cycles. Using a modified Dean-Stark apparatus, 200 g of silicone oil was depolymerized with only one-tenth the solvent of a one-pot process. The system also efficiently depolymerized both condensation-cured and addition-cured silicone rubbers.

In chemical recycling methods, in addition to conventional acid-catalyzed and base-catalyzed approaches, several distinctive degradation strategies have been developed, including amination, alcoholysis, and steam cracking. In these processes, the lone-pair electrons on the N or O atoms of amines, alcohols, or water attack the silicon centers, thereby promoting Si-O bond cleavage. Broadly, these methods can be regarded as Lewis-base-mediated processes. However, because of their relatively weak basicity, these reagents generally cannot induce the anionic back-biting reaction required for efficient polysiloxane depolymerization.

**Figure 7:**
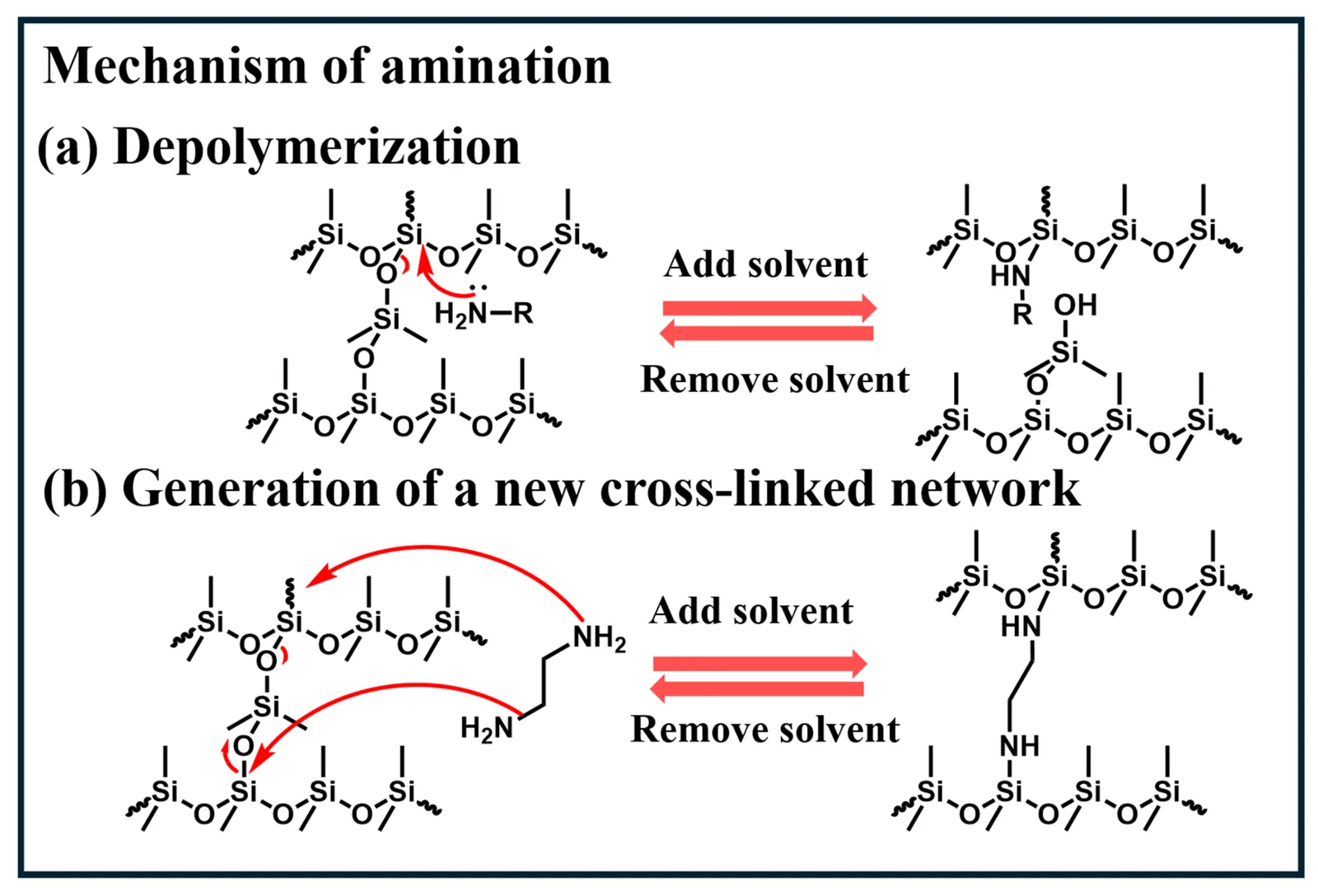
Proposed mechanism of silicone aminolysis [182,183].

Pappas et al. [182,183] achieved the “chemical dissolution” of organosilicon crosslinked materials through amination, followed by regeneration of the crosslinked network after solvent evaporation. They compared the degradation behavior of linear and condensed crosslinked polymers treated with primary, secondary, tertiary, and diamines. The results showed that amines can effectively cleave Si-O bonds at crosslinking sites. In contrast, Si-O bonds in linear polymers exhibited lower reactivity, possibly because the presence of multiple oxygen atoms at the crosslinking sites increased the electrophilicity of the silicon atoms, thereby accelerating their reaction with amines. Pappas et al. further found that the degradation rate was closely associated with the number of reactive hydrogen atoms in the amine and the length of the alkyl chain. Primary amines reacted significantly faster than secondary and tertiary amines, while amines with shorter alkyl chains showed lower steric hindrance and thus higher degradation rates. The crosslinked network could be completely depolymerized within 1–2 days. In contrast, diamines were almost unable to induce effective degradation, likely because their multiple amino groups readily promoted reformation of the crosslinked structure. Furthermore, the aminolysis reaction was reversible, and the crosslinked network was restored after solvent removal, as shown in Figure 7. The aminolysis strategy, in which amines promote the cleavage of Si–O bonds in organosilicon compounds, was subsequently validated by Schimmel [184], Chang [185,186], and co-workers.

Schimmel et al. [184] used gas chromatography to monitor the reactions between diethylamine and polysiloxane crosslinking segments as well as tetrafunctional model compounds. They found that Si-O bond cleavage occurred only at tetrafunctional Si-O sites. Diethylamine was unable to cleave the linear model compounds hexamethyldisiloxane (MM) and octamethyltrisiloxane (MDM), whereas the tetrafunctional compound M_4_Q underwent aminolysis within 78 h. When a diethylamine/KOH mixed reagent was used, the depolymerization time was shortened to 4 h.

Chang et al. [185] found that room-temperature depolymerization of organosilicon crosslinked materials could be achieved in amine/alcohol mixed solvents in the presence of strong bases. In polar solvents, silicone rubber underwent nucleophilic cleavage by alcohols or amines to yield dimethylcyclosiloxane cyclic compounds. In contrast, in weakly polar media such as toluene, polymers with higher molecular weights were predominantly recovered. The DMC cyclic compounds were generated through back-biting reactions of active chain ends. The back-biting rate depended on the mobility of polymer chain segments and the ability of the solvent to dissolve the active end groups, with cyclization of polar end groups being more favorable in polar solvents.

Chang et al. [186] further investigated the aminolysis capability of ethylenediamine toward condensation-cured silicone rubber in an ethanol solution containing KOH. In weakly polar toluene, the main aminolysis products were linear PDMS chains bearing hydroxyl and ethoxy terminal groups, with an average molecular weight of 1.5 × 10^4^. In the more polar solvent tetrahydrofuran, the degradation rate was significantly accelerated, accompanied by the formation of large amounts of DMC cyclic compounds. For different KOH/alcohol solutions, the dissolution time decreased in the following order: ethanol > 1,4-butanediol > ethylene glycol > glycerol.

Petrus et al. [187] developed a solvothermal alcoholysis method for the depolymerization of silicone rubber using fatty alcohols under both catalyst-free and catalytic conditions. Under catalyst-free conditions, alkoxy-terminated PDMS liquid oligomers were obtained at 180–200 °C after 16–18 h. When the reaction temperature was increased to 220–240 °C, further depolymerization occurred, producing R-(OSiMe_2_)_x_-OR species, where x = 1, 2, 3, and 4, denoted as P_1_, P_2_, P_3_, and P_4_, respectively. In the presence of a Mg-Na/K binary catalyst, the reaction at 220 °C for 2 h afforded optimal product yields of 79% P_1_ and 17% P_2_.

Torgunrud et al. [85] investigated the depolymerization of PDMS with diols under basic conditions and found that 2-methyl-2,4-pentanediol could cap Si-O segments. When a non-catalytic amount of this compound was added, PDMS was converted into alkoxy monomers. However, the entire system remained in dynamic equilibrium; therefore, continuous distillation was required to remove the products in situ and drive the formation of alkoxy monomers.

Zhang et al. [84] employed alcohols such as nerol, geraniol, and linalool to depolymerize waste silicone rubber into liquid siloxane derivatives. Different alcohols led to different ratios of linear oligomers and dimethylcyclosiloxane cyclic compounds in the depolymerization products. Geraniol produced a higher proportion of alkoxy-terminated linear PDMS oligomers, whereas linalool yielded a higher content of DMC cyclic compounds. When the resulting liquid siloxanes were used as additives in nitrile rubber/silica composites, both tensile strength and elongation at break were improved. When used as plasticizers, they enhanced the processability and low-temperature resistance of the composites.

Base-catalyzed methods generally require relatively low catalyst loadings. Through nucleophilic attack and silanolate anion back-biting mechanisms, these methods can selectively produce cyclic oligomers with relatively high selectivity. However, in the later stage of the reaction, an excessively high base concentration may induce the cleavage of Si–C bonds in the side chains of silicone polymers, leading to the formation of silicone resin structures containing T and Q units. At present, the degradation of silicone polymers using Lewis bases, ionic liquids, and other catalytic systems containing less sterically hindered nucleophilic species has become one of the current research hotspots. These systems are characterized by mild reaction conditions and high yields of DMC cyclic oligomers. Current studies mainly focus on increasing the effective concentration and controlled release of nucleophilic species within the catalytic system.

### 2.3. Comparison of Selected Research on Silicone Polymer Recycling

Currently, recycling strategies for organosilicon polymers can be broadly classified into two categories: physical recycling methods and chemical recycling methods. For physical recycling methods, the relevant literature is systematically reviewed and categorized according to conventional physical recycling methods, irradiation-assisted technologies, and ultrasound-assisted technologies. Key information, including the literature reference, reaction conditions, product yields, and selectivities, is summarized in Table 1. For chemical recycling methods, representative studies are comparatively discussed in the order of thermal pyrolysis, acid-catalyzed degradation, and base-catalyzed degradation. The corresponding literature data are compiled in the same format and presented in Table 2.

### 2.4. Analysis of Advantages and Disadvantages of Silicone Polymer Recycling Methods

Current recycling strategies for silicone polymers can generally be classified into two categories: physical recycling and chemical recycling. Physical recycling mainly includes mechanical comminution, irradiation, and ultrasonic treatment, whereas chemical recycling primarily involves thermal pyrolysis, acid-catalyzed depolymerization, alkali-catalyzed depolymerization, Lewis acid catalysis, Lewis base catalysis, and solvolysis-based methods. The advantages and limitations of these technologies are summarized as follows.

(1) Mechanical comminution: Mechanical comminution is relatively simple to operate and is suitable for applications with low requirements for material strength and performance. Its major limitation is that the surface of the pulverized silicone rubber particles contains few reactive functional groups, resulting in weak interfacial interactions with the surrounding matrix. In addition, silicone rubber fillers are difficult to separate from modified materials such as asphalt and cement, which further restricts the recyclability of mechanically recovered silicone rubber.

(2) Irradiation and ultrasonic treatment: Irradiation and ultrasonic treatment mainly use physical energy input to generate free radicals, thereby promoting surface crosslinking or surface modification of silicone rubber. These methods, however, do not enable depolymerization of silicone polymers into small molecules or efficient recovery of fillers. They are therefore more commonly used for surface treatment, modification, and accelerated aging of silicone rubber, with the resulting materials mainly applied in environments involving extreme temperatures or radiation exposure.

(3) Thermal pyrolysis: Thermal pyrolysis was initially used to evaluate the thermal stability of polymeric materials. It requires no catalyst and represents one of the earliest and most established methods for the degradation and recovery of silicone polymers. This method can depolymerize polyorganosiloxanes into cyclic small molecules. Nevertheless, thermal pyrolysis also induces radical-mediated side-chain cleavage in addition to main-chain depolymerization, generating gaseous hydrocarbons, carbonaceous residues, and other byproducts. These side reactions significantly reduce the yield and selectivity of cyclic products. Moreover, the high energy consumption of thermal pyrolysis is inconsistent with the current demand for green and low-carbon recycling technologies.

(4) Protonic acid-catalyzed depolymerization: Strong protonic acids can efficiently cleave Si-O-Si bonds under relatively mild conditions, leading to the depolymerization of polyorganosiloxanes with only a small amount of catalyst. However, strong acids are highly corrosive to equipment and create additional challenges associated with waste acid treatment. The degradation products are usually mixtures of cyclic and linear compounds, which complicates downstream separation. In addition, when silicone rubber contains alkaline fillers such as CaCO_3_, or Mg(OH)_2_, the acid reacts with these fillers, resulting in increased acid consumption and greater difficulty in filler recovery.

(5) Lewis acid catalysis: Lewis acid catalysis is an emerging and actively investigated approach for silicone polymer depolymerization. Lewis acids can activate and cleave Si–O–Si bonds through their electron-deficient centers. This method generally requires a low catalyst loading and can operate under relatively mild conditions, enabling the conversion of polysiloxanes into silane monomers. Its main limitation lies in the requirement for large amounts of non-catalytic end-capping agents, such as halogen-containing reagents or alkoxy reagents. During subsequent hydrolysis and condensation, the resulting silane monomers may generate considerable quantities of byproducts, including hydrohalic acids and alcohols.

(6) Strong alkali-catalyzed cleavage: Strong alkali-catalyzed cleavage requires only a small amount of catalyst, and its reaction mechanism is more clearly understood than that of acid-catalyzed depolymerization. The process mainly involves nucleophilic attack on silicon atoms followed by intramolecular back-biting of silanolate anions. Compared with other methods, strong alkali catalysis generally exhibits higher selectivity toward cyclic dimethylsiloxane oligomers. The principal drawback is that, in the late stage of the reaction, the increased concentration of strong base may induce cleavage of Si-C bonds in the side chains of organosilicon polymers. This can lead to the formation of silicone-like crosslinked structures containing T and Q units, thereby decreasing the yield of cyclic dimethylsiloxane oligomers.

(7) Lewis base catalysis: Lewis base catalysis also requires only a small amount of catalyst. Low-steric-hindrance Lewis bases can attack Si-O-Si bonds, and the reaction mechanism is generally consistent with that of strong base-catalyzed depolymerization. This strategy also shows high selectivity toward cyclic dimethylsiloxane oligomers. Current research on Lewis base catalysis mainly focuses on two systems: fluorinated quaternary ammonium ionic liquids and alkali metal salts. Fluorinated quaternary ammonium ionic liquids can dissociate almost completely, providing high concentrations of nucleophilic species. However, they suffer from limitations such as structural instability of the cation and the possible generation of toxic compounds, such as tributylamine, during thermal decomposition. By contrast, alkali metal salts exhibit limited dissociation in organic phases and therefore provide insufficient nucleophile concentrations. Consequently, phase-transfer catalysts, including multidentate ligands, are often required to enhance the concentration of active nucleophilic species in the organic phase.

(8) Aminolysis and alcoholysis: Aminolysis can efficiently degrade crosslinking sites containing Si-O bonds in condensation-cured silicone rubbers. However, it is less effective for cleaving Si-C-based crosslinking sites in addition-cured silicone rubbers or thermoset silicone rubbers. At present, aminolysis and alcoholysis alone generally show insufficient depolymerization capability toward organosilicon polymers. They are more commonly used as auxiliary solvents or co-reaction systems and can play a more significant role when combined with strong alkaline catalysts.

Most current studies on silicone polymer recycling technologies primarily focus on depolymerization mechanisms, catalytic efficiency, and target product recovery yields, whereas limited attention has been paid to energy consumption, greenhouse gas emissions, wastewater treatment burdens, and overall resource efficiency within a unified system boundary. Consequently, it remains challenging to rigorously and quantitatively rank the environmental benefits of different recycling pathways. To address this gap, this study develops a qualitative/semi-quantitative comparative framework based on green chemistry principles to evaluate the relative advantages and limitations of different technical routes in terms of reaction conditions, catalyst sustainability, solvent use, by-product formation, closed-loop recycling potential, and industrial applicability, which is summarized in Table 3.

In terms of CO_2_ emissions and environmental impact, mechanical comminution generally exhibits relatively low direct carbon emissions and energy consumption during the recycling of silicone polymers. Its primary advantages lie in its operational simplicity and the absence of complex chemical reagents. However, this approach is essentially a downcycling strategy. Crushed silicone rubber is typically used as a filler in modified materials, making it difficult to convert it back into high-quality silicone feedstocks. In addition, the mechanical properties of the recycled materials tend to deteriorate after repeated recycling cycles. Therefore, although physical crushing can partially alleviate the pressure associated with waste disposal, it does not enable true closed-loop recycling of silicone materials. Moreover, the subsequent disposal of low-value recycled products and the upstream production of new silicone monomers may still lead to considerable energy consumption and CO_2_ emissions.

In contrast, thermal cracking can depolymerize silicone polymers into small molecules, such as cyclic siloxanes. However, this process usually requires harsh reaction conditions, including temperatures above 350–400 °C, reduced pressure, and/or an inert atmosphere, resulting in substantial energy consumption. Although pyrolysis generally avoids the extensive post-treatment associated with large quantities of acid or base catalysts, its product distribution is often broad. Consequently, the yield and selectivity of target cyclic dimethylsiloxanes, as well as the efficiency of subsequent separation processes, remain limited.

Compared to thermal cracking, strong acid-catalyzed depolymerization is typically conducted under milder reaction conditions and requires lower energy input. This method can convert silicone polymers into low-molecular-weight siloxanes with potential for repolymerization, thereby reducing reliance on upstream processes such as silica mining, silicon smelting, and monomer synthesis. As a result, it offers certain advantages in terms of carbon emission reduction. Nevertheless, strong acid systems are still associated with several challenges, including equipment corrosion, difficulties in acid recovery, waste acid treatment, and the control of side reactions.

Similarly, strong base-catalyzed systems can promote the cleavage of siloxane bonds under relatively mild conditions, generating cyclic compounds or oligomers that can re-enter the silicone recycling loop. This contributes to reduced resource consumption and CO_2_ emissions associated with primary silicone production. However, practical applications of strong base systems still require careful consideration of side reactions, the accumulation of non-volatile residues, and the long-term stability of both the catalyst and the reaction system.

In recent years, Lewis acid/base systems and other emerging catalytic strategies have attracted increasing attention in the chemical recycling of silicones. Some of these systems can achieve rapid depolymerization at room temperature or under mild conditions, producing cyclic siloxanes, chlorosilanes, or other reusable organosilicon small molecules in high yields. These features indicate their potential for reducing energy consumption and improving recycling efficiency. However, several challenges remain before large-scale industrial implementation can be realized, including catalyst regeneration and reuse, reagent cost and safety, compatibility of the product streams with existing organosilicon supply chains, and the effects of fillers and crosslinked structures in complex waste silicone rubber on reaction efficiency.

Regarding the recycling of the fillers, during mechanical comminution, fillers, additives, and the silicone rubber matrix generally remain together in the ground rubber powder [75] and are subsequently incorporated into new materials [94]. However, the direct introduction of these multicomponent particles may lead to inadequate dispersion and poor interfacial compatibility with the new polymer matrix, thereby limiting the performance of the resulting composites.

During thermal cracking, by contrast, inorganic fillers are generally concentrated in the solid residue, while hydroxide fillers may undergo dehydration and phase transformation. Under appropriately controlled conditions, silicone-containing waste may also be converted into ceramic precursors or value-added ceramic products [140], such as silicon carbide.

At present, studies on the efficient separation and recovery of fillers are mainly focused on alkaline-catalyzed depolymerization. Industrial silicone rubber formulations commonly contain acid-reactive fillers or additives, such as CaCO_3_ and Mg(OH)_2_, which may consume acidic catalysts, generate inorganic salts, or complicate downstream separation. Compared with acid-catalyzed processes, appropriately designed alkaline systems may therefore better preserve the chemical composition and structural integrity of certain fillers. In particular, catalytic systems involving amines and tetramethylammonium hydroxide [167] can promote the selective depolymerization of the silicone matrix and facilitate its separation from the inorganic fillers. Residual alkaline species adsorbed on the filler surfaces can subsequently be removed through controlled heating, followed by filtration, washing, and drying, yielding relatively clean fillers that may be reused in new material formulations. Nevertheless, the recovery behavior and structural stability of fillers depend strongly on their composition, surface treatment, and the specific reaction conditions employed. Research on filler separation and recovery in other emerging silicone recycling routes remains limited. Therefore, developing efficient, selective, and economically viable strategies for the recovery and reuse of fillers represents an important direction for both current and future research.

### 2.5. Technology Readiness Level (TRL) of Silicone Polymer Recycling Technologies

Due to the different development stages of various silicone recycling technologies, their applicable scenarios, recycling efficiency, and the quality of recovered products vary significantly. Based on available literature reports and publicly accessible technical information, this study provides a comprehensive assessment of existing silicone recycling technologies and classifies their development stages according to the Technology Readiness Level (TRL) [188,189,190,191].

Among the currently available approaches, mechanical comminution [75,91,92] is considered one of the most technologically mature methods for silicone recycling. This method involves relatively simple equipment, convenient operation, low requirements for operator expertise, and the capability for large-scale processing of silicone waste. However, the recycling process mainly relies on mechanical size reduction for material reuse, resulting in recovered products with relatively limited performance and added value. Therefore, it is primarily suitable for applications where strict requirements on the properties of recycled materials are not necessary. Its technology maturity is estimated to reach TRL 7–8.

In contrast, emerging technologies such as irradiation [87], ultrasonic treatment [77,103], and supercritical fluid-assisted processes [78] have mainly been investigated for aging regulation, performance modification, and structural manipulation of silicone rubbers. These approaches are currently regarded as auxiliary technologies for silicone degradation and modification rather than mature recycling routes. Although they have demonstrated certain potential at the laboratory scale, their practical implementation is restricted by factors including equipment cost, energy consumption, and challenges associated with scale-up. Therefore, their current technology maturity is estimated to be approximately TRL 2–3.

Thermal cracking technology can be traced back to the 1940s [118], representing one of the earliest approaches investigated for silicone materials. In its early development stage, thermal pyrolysis was mainly employed to evaluate the thermal stability and degradation behavior of polysiloxanes. Although the fundamental principles and pyrolysis mechanisms are relatively well understood, its application in industrial-scale silicone polymer recycling remains limited due to challenges including high energy consumption and poor product selectivity [119]. Therefore, the current TRL of thermal cracking is estimated to be approximately TRL 4–6.

Acid- and base-catalyzed cracking technologies, such as those using H_2_SO_4_ or KOH, have a relatively long research history, with studies dating back to the 1960s [46,147]. These methods have gradually developed into important approaches for recovering silicone monomers and low-molecular-weight siloxanes from silicone waste. In particular, alkaline catalytic processes can convert silicone polymers into cyclic siloxanes (DMC, mainly D_4_-D_6_) through Si-O-Si bond cleavage, silanol-mediated rearrangement, and intramolecular back-biting reactions, followed by ring-opening polymerization to produce downstream silicone products. Due to their well-established reaction mechanisms and relatively mature process pathways, alkaline cracking technologies possess considerable potential for industrial implementation, with an estimated maturity level of TRL 6–7. Nevertheless, compared with highly developed polymer recycling technologies, such as those for plastics and polyethylene terephthalate (PET), silicone recycling still faces challenges associated with feedstock collection, process optimization, product purification, and economic feasibility. Acid-catalyzed processes generally exhibit lower maturity than alkaline methods due to issues including equipment corrosion, side reactions, and acid waste treatment requirements, with an estimated maturity level of TRL 5–6.

Recently, the controlled depolymerization of silicone polymers under mild conditions using Lewis acid/base catalysts [73,192] has attracted increasing attention. This approach offers advantages including mild reaction conditions, high selectivity, and the potential for high-value recovery of silicone monomers. However, current studies remain predominantly limited to laboratory-scale investigations, and significant challenges remain before industrial implementation. Therefore, the technology maturity of Lewis acid/base catalytic depolymerization is estimated to be approximately TRL 4–5.

Furthermore, continuous catalytic cracking of silicone polymers using tubular reactors loaded with supported solid acids [157] or bases [173] represents a promising strategy for achieving scalable and continuous recycling processes. However, this technology is still mainly at the experimental validation stage, with only limited patent reports concerning process development. A mature industrial recycling system has not yet been established, and its current technology maturity is estimated to be approximately TRL 4–5. More details are shown in Table 4.

## 3. Conclusions

Considerable progress has been made in both academia and industry in the depolymerization and recycling of silicone polymers, leading to promising outcomes. The degradation and recycling of silicone polymers can contribute to reducing CO_2_ emissions and mitigating their environmental impact.

Overall, the various silicone polymer recycling methods described in this review each present distinct application scenarios and inherent limitations. In practice, the selection of a suitable process requires a balanced consideration of multiple factors, including target product value, recycling efficiency, energy consumption, and technical maturity. Specifically, mechanical comminution stands out for its operational simplicity and low cost, making it a viable option for producing fillers in applications where high product performance is not critical and the primary goal is resource utilization. Both acid-catalyzed and alkali-catalyzed methods enable effective separation of fillers from the polymer matrix. Acid catalysis is more favorable for recycling high-value organosilicon monomers, whereas alkali catalysis exhibits superior performance in producing DMC cyclic oligomers and separating alkaline fillers such as CaCO_3_ and Mg(OH)_2_. Ultrasound- and radiation-assisted methods are primarily applicable to the degradation treatment of silicone rubbers that have undergone aging under extreme thermal or radiation environments. However, their broader applicability and industrial scalability remain constrained by high energy demands and significant equipment investment. Pyrolysis, despite its considerable energy consumption, offers irreplaceable advantages in the production of high-value inorganic materials, such as silicon carbide (SiC) and ceramic precursors.

Therefore, for practical applications, it is neither meaningful nor advisable to rank these technologies by absolute superiority. Instead, a comprehensive evaluation should be performed based on specific recycling objectives and operational boundary conditions. Future research should prioritize the establishment of a standardized evaluation framework and integrate life-cycle assessment (LCA) to enable a holistic comparison of the economic viability and environmental sustainability of different technological routes. Such efforts will be essential to facilitate the transition of silicone rubber recycling technologies from laboratory-scale research to viable industrial implementation.

Current studies on the degradation of organosilicon polymers have mainly focused on process optimization, whereas the fundamental principles and molecular-level mechanisms governing organosilicon recovery remain insufficiently understood. This knowledge gap limits the rational design and further development of efficient recycling technologies. Future research on organosilicon recovery may therefore focus on the following directions.

(1) Model compounds should be used in combination with in situ and real-time monitoring techniques to clarify the molecular mechanisms of silicone polymer degradation and recovery. Spectroscopy, mass spectrometry, chromatography, and other complementary analytical methods can provide mechanistic information on bond cleavage, intermediate formation, product evolution, and side reactions.

(2) The efficient depolymerization of silicone polymers under mild conditions using greener catalysts and environmentally benign solvents represents a key direction for future development. Particular attention should be given to catalytic systems with high activity, high selectivity, low toxicity, good recyclability, and compatibility with complex silicone waste streams.

(3) Selective recovery strategies should be developed to convert mixed cyclic dimethylsiloxane oligomers into individual cyclic products. Coupling depolymerization reactors with distillation, membrane separation, or other separation units may provide a promising route for the selective recovery of target cyclic monomers. This direction remains insufficiently explored and has considerable potential for improving the value and efficiency of silicone polymer recycling.

## Figures and Tables

**Table 1:** Comparison of Selected Research on Physical Recycling Methods for Silicone Polymers.

Reference	Materials	Experimental Conditions	Results
[74]	Silicone rubber powder	10,000 rpm mixing for 18 s; hot pressing at 160 °C and 5 MPa for 600 s; post-curing at 120–180 °C for 6 h.	At a content of 10%, the wear rate of the material decreased by 29%, while the average amplitude of friction-induced vibrations was reduced by 35.5%.
[75]	Silicone rubber powder with a particle size greater than 50 μm	(1) Rubber mastication; (2) Incorporation and homogenization of recycled powder; (3) Addition of crosslinking agent and final homogenization.	At a loading of 60 phr, the compression deformation increased by 22% compared with that at 0 phr, while the maximum stress and elongation at break decreased by 45% and 25%, respectively.
[87]	Silicone rubber/EPDM rubber blend (1:1)	The Gamma chamber has an irradiation volume of one litre with a dose rate of 3 KGy·h^−1^. The applied dose was 25 Mrad.	The elongation at break decreased with increasing crosslinking density but remained above 50% of the initial value.
[91]	Discarded silicone rubber insulators (from 0.3 to 4.0 mm)	Immersion in H_2_O_2_ or KOH solution at room temperature for 24 h; washing and surface modification; subsequent incorporation into cement mortar.	At a replacement level of 15%, the incorporation of H_2_O_2_- and KOH-modified silicone rubber increased the compressive strength by 10.56% and 12.44%, respectively.
[94]	Crushed liquid silicone rubber (LSR)	Incorporation of recovered powder into liquid silicone rubber, followed by conventional injection molding.	At a recycled powder loading of 40%, the tensile strength and elongation at break of the recycled liquid silicone rubber decreased by 16.6% and 12.6%, respectively.
[115]	Silicone rubber sheet (length: 15 cm; thickness: 0.55 mm; density: 1.20 g·cm^−3^; average molecular weight between crosslinks: 2.25 × 10^3^ g·mol^−1^)	Al electrodes: 20 mm diameter; Vacuum pressure: <5 Pa; AC voltage: 3 V_rms_; frequency range: 10^−2^ (or 10^−3^)−10^4^ Hz; Temperature: 100–200 °C; Surface potential decay: needle-plate electrode system, −12 kV (needle)/−9 kV (plate), 50 °C; Charging time: 2 min.	Gamma irradiation enhanced instantaneous polarization, reduced the thermal expansion coefficient, and altered trap energy and DC conductivity.
[116]	High-temperature vulcanized (HTV) silicone rubber	UVA-340 nm; UVB-313 nm; Light source distance from silicone rubber: 50 mm; Irradiance: 55 mW.·cm^−2^.	After 96 h of Soxhlet extraction with cyclohexane, the extract was found to contain cyclic siloxanes D_3_-D_19_ and linear oligosiloxanes L_8_-L_17_.
[117]	Phenyl silicone rubber (containing 10% fumed silica)	ECR power: 60–100 W; Chamber pressure: 10^−4^ Torr; AO energy: 5 eV; AO flux: 1.5 × 10^16^	Following oxygen plasma treatment, the silicone rubber exhibited increased crosslink density and modulus, accompanied by reductions in elongation at break and flexibility.

**Table 2:** Comparison of Selected Research on Chemical Recycling Methods for Silicone Polymers.

Reference	Materials	Experimental Conditions	Results
[118]	PDMS	Temperature: 350–400 °C; Atmospheric pressure; Nitrogen atmosphere.	D_3_ selectivity: 44%; D_4_ selectivity: 24%.
[135]	Waste silicone rubber insulator	Temperature: 300–600 °C.	D_3_ selectivity increased from 1.7% (300 °C) to 78.8% (600 °C).
[153]	Block copolymers consisting of poly(tetramethylphenylene) segments and PDMS segments	48%HF; Temperature: 30 °C; Reaction time: 40 h	The crystallinity of the polymer increased from 20% to 98%
[89]	Hydroxyl-terminated polydimethylsiloxane(PDMS-OH, Mn = 550g·mol^−1^)	3 times the amount of benzoyl fluoride; 5 mol% Zn(OTf)_2_; Temperature: 100–150 °C; Reaction time: 5 h	Dimethyldifluorosilane yield: 78%; 1,2-difluoro-1,1,2,2-tetramethyldisiloxane yield: 21%
[146]	Hydroxyl-terminated polydimethylsiloxane(PDMS-OH, Mn = 550g·mol^−1^)	3 times the amount of benzoyl fluoride; 1 mol% Zn(OTf)_2_; Temperature: 130 °C; Reaction time: 1 h	Dimethyldifluorosilane yield: 62%; 1,2-difluoro-1,1,2,2-tetramethyldisiloxane yield: 28%
[73]	Methyl-terminated polydimethylsiloxane (n = 70)	1.1 times the amount of Si-O bonds of BCl_3_; 0.5 mol% GaCl_3_; Temperature: 40 °C; Reaction time: 30 min	98% yield of dimethyldichlorosilane; 2% yield of 1,2-dichloro-1,1,2,2-tetramethyldisiloxane
[155]	Recycled silicone rubber powder	H_2_SO_4_/Al_2_O_3_-TiO_2_; Temperature: 130 °C; Reaction time: 4h.	A total of 250 g of DMC was obtained from 400 g of recycled silicone rubber powder.
[166]	Room-temperature-vulcanized (RTV) silicone rubber	Solvent: toluene; 0.08 mol% KOH; Reaction conditions: 160 °C, 50 mmHg, 3 h.	DMC yield: 65%; D_3_ selectivity: 13.8%; D_4_ selectivity: 71.5%; D_5_ selectivity: 10.4%; D_6_ selectivity: 1.3%.
[167]	High-temperature-vulcanized (HTV) silicone rubber	N(CH_3_)_4_OH, KOH, a mixed solvent of diethylamine, methanol, and hexane; Distilled for 3 h at 170 °C and 30 mmHg.	Clean SiO_2_ filler was recycled, with a DMC yield of 84%.
[170]	Polymethylphenylsiloxane	TBAF; Solvent: THF; Temperature: room temperature; Reaction time: 24h.	D_Ph_^4^ yield: 73.2%, D_Ph_^5^ yield: 22.5%.
[173]	Hexamethylcyclotrisiloxane	KF/Al_2_O_3_ solid catalyst; Temperature: 400 °C	Me_2_Si(OMe)_2_ yield: 94%.

**Table 3:** Analysis and Evaluation of Different Silicone Polymer Recycling Technologies within a Green Chemistry Framework.

Recycling Technologies	Reaction Conditions	Catalyst Sustainability	Solvent Use	By-Product Formation	Closed-Loop Recycling Potential	Industrial Applicability
Mechanical comminution	Room temperature and atmospheric pressure	No catalyst	No solvent use	Hardly	Hardly	High feasibility for industrial-scale application
Irradiation, ultrasonic and supercritical fluid technologies	Radiation/ultrasound environment, high energy consumption	Little or no catalyst required	Supercritical fluid will use solvent	Hardly	Hardly	At the laboratory stage
Protonic acid (such as H_2_SO_4_)	100–200 °C, vacuum environment	Catalyst required	A certain amount of solvent is required	Certain amount of by-products generated	Recyclable	Preliminary industrial application basis
Thermal Cracking	High temperature, protective atmosphere and non-atmospheric pressure	Hardly	Hardly	A large amount of by-products is generated.	Hardly	At the laboratory stage
Strong base (such as KOH)	100–200 °C, vacuum environment	Catalyst required	A certain amount of solvent is required	A little by-products generated	Recyclable	Preliminary industrial application basis
Lewis acid/base depolymerization	The reaction conditions are relatively mild compared with strong acid or strong base conditions.	Catalyst required	A certain amount of solvent is required	A little of by-products generated	Recyclable	At the laboratory stage
Solid catalyst	High temperature, protective atmosphere and non-atmospheric pressure	Catalyst required	No solvent use	Certain amount of by-products generated	Insufficient circulation	At the laboratory stage

**Table 4:** TRL of silicone polymer recycling technologies.

Recycling Technologies	Estimate TRL
Mechanical comminution	7–8
Irradiation, ultrasonic and supercritical fluid technologies	2–3
Thermal Cracking	4–6
Protonic acid (such as H_2_SO_4_)	5–6
Strong base (such as KOH)	6–7
Lewis acid/base depolymerization	4–5
Solid catalyst	4–5
TRL Description [188,189,190,191]: Basic research stage (TRL 1–3): Verification of scientific principles TRL 1: This stage involves identifying emerging concepts, potential integration pathways, and anticipated technical challenges based on fundamental theoretical principles. TRL 2: This stage requires further development of knowledge regarding the underlying technologies, materials, and interface interactions. The proposed concept is refined through theoretical analysis and preliminary feasibility assessments. TRL 3: This stage involves establishing the first laboratory-scale prototypes or numerical models to demonstrate the feasibility of the proposed technology. Key parameters are identified, and the fundamental principles are experimentally or computationally validated. Development verification stage (TRL 4–6): From laboratory to real environment TRL 4: This stage involves the development of reduced-scale prototypes integrated with relevant subsystems under laboratory conditions. The technology performance is evaluated through controlled experiments and quantitative key performance indicators (KPIs). TRL 5: This stage involves integrating technological components with supporting subsystems into larger-scale prototypes. Validation is conducted under conditions that closely represent real-world operating environments to demonstrate technical feasibility beyond laboratory settings. TRL 6: This stage requires full-scale prototype demonstration in a relevant operational environment. System integration, performance evaluation, and reliability testing are conducted to verify the technology’s capability under realistic application conditions. Deployment application stage (TRL 7–9): System maturity and commercialization TRL 7: This stage involves demonstrating the complete system prototype in an actual operational environment with all necessary supporting systems. Performance validation is performed under realistic operating conditions, incorporating user feedback and operational experience. TRL 8: This stage represents the completion and qualification of the technology system through comprehensive testing, verification, and demonstration. The final system achieves reliable operation under expected conditions with completed quality assurance and performance validation. TRL 9: This stage represents full-scale deployment and successful long-term operation of the technology. The system demonstrates reliable performance across diverse operational conditions with proven commercial or practical application records.	

## Data Availability

No new data were created or analyzed in this study. Data sharing is not applicable to this article.
